# Structure of *In Vitro*-Synthesized Cellulose Fibrils Viewed by Cryo-Electron Tomography and ^13^C Natural-Abundance Dynamic Nuclear Polarization Solid-State NMR

**DOI:** 10.1021/acs.biomac.1c01674

**Published:** 2022-03-26

**Authors:** Fabien Deligey, Mark A. Frank, Sung Hyun Cho, Alex Kirui, Frederic Mentink-Vigier, Matthew T. Swulius, B. Tracy Nixon, Tuo Wang

**Affiliations:** † Department of Chemistry, Louisiana State University, Baton Rouge, Louisiana 70803, United States; ‡ Department of Biochemistry and Molecular Biology, Pennsylvania State University, University Park, Pennsylvania 16802, United States; § National High Magnetic Field Laboratory, Tallahassee, Florida 32310, United States; ∥ Department of Biochemistry and Molecular Biology, Pennsylvania State University, Hershey, Pennsylvania 17033, United States

## Abstract

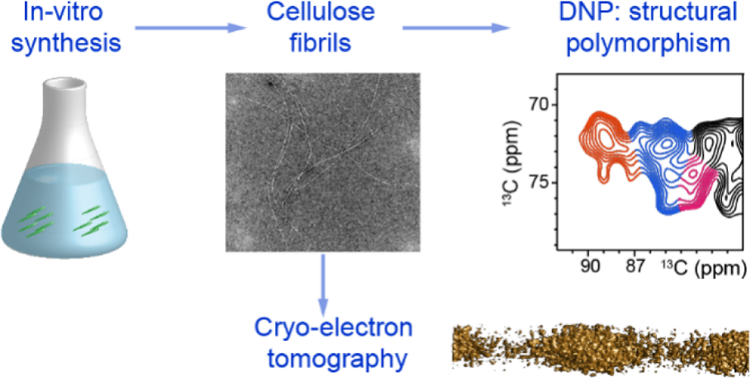

Cellulose, the most abundant biopolymer, is a central source for renewable energy and functionalized materials. *In vitro* synthesis of cellulose microfibrils (CMFs) has become possible using purified cellulose synthase (CESA) isoforms from *Physcomitrium patens* and hybrid aspen. The exact nature of these *in vitro* fibrils remains unknown. Here, we characterize *in vitro*-synthesized fibers made by CESAs present in membrane fractions of *P. patens* over-expressing CESA5 by cryo-electron tomography and dynamic nuclear polarization (DNP) solid-state NMR. DNP enabled measuring two-dimensional ^13^C–^13^C correlation spectra without isotope-labeling of the fibers. Results show structural similarity between *in vitro* fibrils and native CMF in plant cell walls. Intensity quantifications agree with the 18-chain structural model for plant CMF and indicate limited fibrillar bundling. The *in vitro* system thus reveals insights into cell wall synthesis and may contribute to novel cellulosic materials. The integrated DNP and cryo-electron tomography methods are also applicable to structural studies of other carbohydrate-based biomaterials.

## Introduction

Cellulose is the most abundant biopolymer on Earth. It comprises the majority of plant biomass and serves as a major reservoir of renewable energy and functional biomaterials.^1−4^ In the primary and secondary cell walls of plants, cellulose exists in the form of crystalline microfibrils, providing support and rigidity to the cells.^5^ Chemically, each elementary cellulose microfibril (CMF) (3–4 nm across) is presumably assembled by 18 chains of β-1,4-glucans held together by numerous hydrogen bonds.^6,7^ Elementary microfibrils further associate to form large bundles that are 10–20 nm across, which happens particularly often in secondary plant cell walls.^8^

In the plant cell, each individual glucan chain is produced by the cellulose synthase (CESA) proteins located at the plasma membrane, from uridine diphosphate-glucose (UDP-α-d-Glc) substrate.^9,10^ CESA units are themselves arranged in a larger hexagonal structure called the CESA complex (CSC). For decades, the exact number of glucan chains in a microfibril has remained elusive. Initially, a 36-chain model was proposed based on the assumption that each CSC might have a hexamer of hexamer organization.^11^ However, diffraction and spectroscopic data supported smaller models with either 24 or 18 chains in each microfibril.^12−14^ Most recent studies suggest that each lobe typically contains three CESAs.^6,15−17^ Consequently, each CSC could polymerize up to 18 glucan chains at once,^18,19^ making the 18-chain arrangement the best-accepted model. Thereafter, spatial proximity between the newly synthesized chains allows the formation of CMFs due to electrostatic interactions (hydrogen bonding and van der Waals forces) relayed by hydroxide groups,^20^ followed by a bundling process into larger fibrils.^21^

To rationally engineer plants and tailor cellulose production to fulfill current needs for energy and material, an in-depth understanding of cellulose biosynthesis and assembly is needed. Previously, *in vitro* cellulose synthesis was reported using plant membrane fractions of blackberry, mung bean, hybrid aspen, and tobacco.^22−25^ We have also successfully developed *in vitro* replication of cellulose biosynthesis starting from a UDP-glucose medium^26^ and the solubilized protein from microsomes of *Physcomitrium patens* overexpressing CESA5 (or purified CESA5 or poplar CESA8 that were expressed in Pichia) purified and reconstituted into proteoliposomes.^27,28^ Because linkage analysis confirmed the synthesis of mostly β-1,4-glucans chains, two questions have arisen. How are these *in vitro*-synthesized cellulose fibers assembled? Can the assembled fibers fully replicate the structure of microfibrils present in native cell walls? To begin answering these questions, we combined cryo-electron tomography (CET) and solid-state NMR to characterize the structure of *in vitro* fibers on the nanoscale and atomic levels, respectively. Achieving the solid-state NMR results required a 10-fold scaling up of the previously reported reaction protocol^26^ and the use of magic-angle spinning (MAS) dynamic nuclear polarization (DNP) to enhance the NMR signal.

Recently, multidimensional solid-state NMR techniques have shown their capability of revealing the molecular structure of cellulose and its interactions with other biopolymers (such as hemicellulose, pectin, and lignin) in native plant cell walls and carbohydrate-based materials.^29−33^ By coupling ^13^C labeling of samples and high-field NMR, seven types of glucose units were consistently identified in the CMFs across the cell walls of a variety of plant genera, including *Arabidopsis*, *Brachypodium*, maize, rice, switchgrass, poplar, eucalyptus, and spruce.^34−36^ None of these glucose units follow the ^13^C chemical shifts of the bulk allomorphs, Iα and Iβ structures,^37^ revealing a substantially deviated structure of cellulose when placed in the native context. However, the expected signals of Iα and Iβ allomorphs have been recently observed in cotton, indicating that model crystal structures are only possible in highly crystalline cellulose with large crystallites.^38^ To apply NMR to reveal the structure of *in vitro* CMF, the methodology employed in previous plant studies must overcome two major challenges: the limited amount of biomaterial that can be obtained *in vitro* and the difficulty in ^13^C-labeling these fibers. Here, we employ MAS-DNP to vastly enhance the NMR sensitivity and eliminate the need for isotope enrichment. High-resolution data provide both qualitative and quantitative information about *in vitro*-synthesized CMFs.^39−43^

Subtomogram averaging of particles obtained *via* CET of *in vitro*-synthesized fibers showed them to contain two interwoven fibers, each about the size of an 18-chain CMF and of similar dimensions to the CET-based structure for CMF in cell walls of *Arabidopsis*(44,45) and onion.^46^ For *Arabidopsis*, using Amira software to model fibers and measure distances, CMF with three types of cross-sectional areas were observed.^44,45^ One type with a 3.5 nm diameter was circular, those of 5.0–5.5 nm were slight ovular extensions of the smaller circular shape, and those of 9–10 nm were oval with dimensions consistent with two adjacent smaller CMFs. Removing matrix materials reduced the larger ovals to 7 nm in diameter. In the onion study, rather than using a simple ruler, a full-width-at-half-maximum (FWHM) approach was used to account for edge distortions caused by birefringence due to imaging cell walls at defocus.^46^ The width of onion CMFs determined using this approach ranged between 5.3 and 6.3 nm. Consistently, the FWHM diameter values for *in vitro*-synthesized fibers that we describe below vary from 4.5 to 6.5 nm depending upon where along the fiber the size is measured.

Two-dimensional (2D) ^13^C–^13^C correlation spectra enabled by MAS-DNP showed that the *in vitro* CMF largely retained the structural features of those microfibrils in intact *Arabidopsis* cell walls. Spectral deconvolution and intensity integration of CMF spectra allow comparing the *in vitro* fibers to previously proposed microfibril structures, with good agreement with the 18-chain arrangement in the microfibril cross-section.^47^ The extensive cross-peaks of a spin-diffusion-based 2D spectrum also allow us to detail the conformers constituting CMF. These results not only shed light on the structure of elementary CMFs but also present a novel strategy for analyzing the high-resolution structures of unlabeled biomaterials.

## Experimental Section

### *In Vitro* CMF Sample Preparation

**Figure 1:**
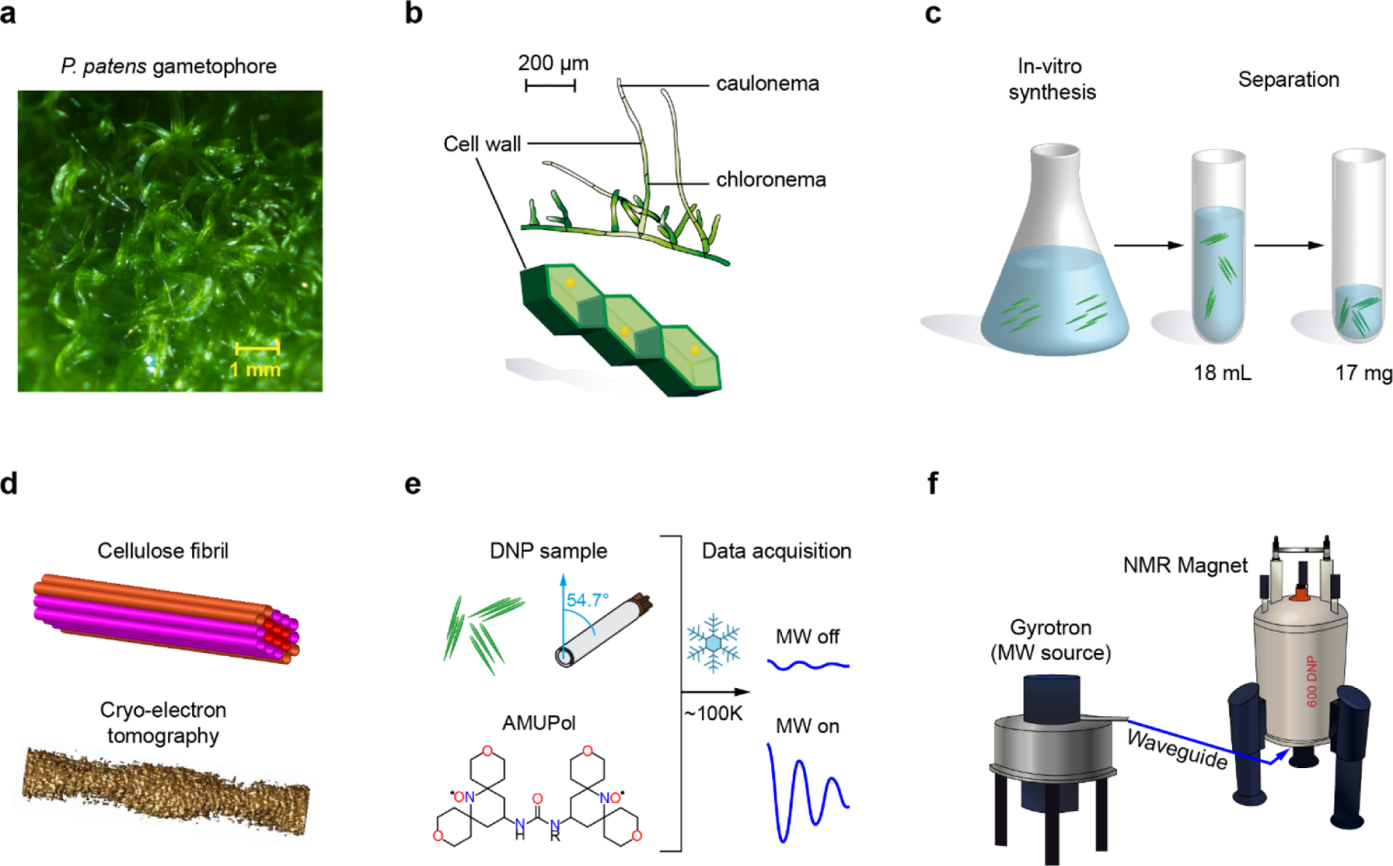
*In vitro* synthesis of cellulose fibrils for structural analysis. (a) *P. patens* moss gametophores viewed by microscopy. (b) *P. patens* cells in the protonema stage and their delimitation by polysaccharide-built cell walls. (c) *In vitro* synthesis of cellulose from UDP-glucose by the microsomal membrane protein fraction containing overexpressed CESA5. (d) Synthesized fibrils viewed by CET. (e) DNP samples and experimental conditions. The fibrils are mixed with AMUPol and packed in a MAS rotor. At cryogenic temperature, the NMR sensitivity will be enhanced when the microwave (MW) is on. (f) MAS-DNP instrument with a 395 GHz gyrotron generating microwave and a 600 MHz NMR magnet.

CMFs were produced *in vitro* following a method previously described,^26^ using the moss *P. patens*(48) overexpressing HA-tagged *P. patens* CESA5. In Figure 1a,b, the growth stages of moss are schematically represented: gametophores will grow from the protonema, which contains two types of elongated cells: chloronema for photosynthesis and caulonema for substrate colonization and nutrient acquisition.^48^ To provide sufficient *in vitro*-synthesized cellulose, 10 membrane preparations were combined and incubated for 24 h at room temperature in the reaction buffer containing 20 mM cellobiose (Figure 1c). For the negative control, 20 μL of the microsomal protein fraction was incubated in buffer lacking UDP-glucose and cellobiose. After incubation, the presence or absence of microfibrils was assessed by placing 3.5 μL from each incubation on carbon-coated copper grids, negatively stained with 0.75% uranyl formate and imaged using an FEI Tecnai 12 Spirit Biotwin transmission electron microscope [FEI; 120 kV; 6.3 mm spherical aberration (Cs); 4k × 4k eagle CCD camera]. The negative control showed no fibers, but they were abundantly present in the experimental sample (Figure S1). To concentrate the microfibrils, 18 mL of the *in vitro* product was centrifuged at 50,000 rpm for 20 min in an Optima Max ultracentrifuge (Beckman Coulter, USA) using a rotor (TLA-100.3) and then discarding the supernatant. The pellet was resuspended in 10 μL of 100 mM MOPS buffer (pH 6.8). The wet weight of the fibers was about 17 mg.

### Subtomogram Averaging

The synthesized CMFs were vitrified by plunge-freezing into liquid ethane using a Vitrobot (FEI), followed by data collection in a Titan Krios system (FEI; 300 kV) using a K3 detector (Gatan) (Figure 1d). Tilt movies of ten frames (5760 × 4096 pixels per frame) were collected dose-symmetrically from 0 to 60° and −60° in 3° increments and processed for motion and contrast transfer function correction using the program Warp.^49^ A small subset of movies was collected with a phase plate, but only those collected without a phase plate were used for subtomogram averaging. The tilt images were aligned and reconstructed into tomograms using IMOD (version 4.12.8) with a rotational tilt-axis of −87°.^50^ Results with the left-handed wrapping of two sub-fibrils are shown, but the actual handedness was not determined. Tomogram reconstruction utilized the default values from the Cryosample.adoc system template except for 2000 × 2000 patches being used for patch tracking and the use of 20 iterations of the SIRT-like filter to enhance the contrast of CMF for fiber annotation. Fiber annotation was performed in 3dmod using open contours placed on straight fibers, avoiding curved ones. Fiber widths were measured with the custom script “sideview-profile-average” written using Ortega.^51^ Model points were added every 126 pixels on each contour using the addModPts command from PEET (version 1.15.0).^52,53^ At bin = 1, this spacing affords non-overlapping subtomograms containing CMF spanning 26.4 nm for averaging.

### MAS-DNP Sample Preparation and Experiments

For atomic-level characterization of the unlabeled CMF material, we employed a matrix-free protocol^54^ to prepare the sample for DNP analysis. Briefly, the CMF material was mixed with a D_2_O/H_2_O mixture (3:1) and 10 mM of bi-nitroxide radical (AMUPol).^55^ The sample was dried in a desiccator at room temperature for about 12 h to remove most D_2_O/H_2_O. Thereafter, 3 μL of D_2_O/H_2_O was added to provide partial moisture to the sample, which has been demonstrated previously to be the key factor in achieving satisfactory DNP enhancement (Figure 1e).^38,54,56−58^

All spectra of the CMF sample were acquired on a 600 MHz (14.1 T) Bruker spectrometer with a 395 GHz gyrotron for microwave generation for DNP enhancement (Figure 1f). The microwave irradiation was 12 W. The sample was packed in a thin-walled 3.2 mm rotor, which was spun at 8 kHz MAS. The temperature at the stator was ∼100 K with microwave irradiation and decreased to 93 K when the microwave was off. The ^13^C chemical shifts are calibrated on the tetramethylsilane (TMS) scale.

For 1D ^1^H–^13^C cross-polarization magic-angle spinning (CP-MAS) experiments, Hartmann–Hahn conditions matched an average ^13^C field of 50 kHz (90 to 110% ramp) with a ^1^H field of 50 kHz during a 1 ms contact time. The DNP buildup time was measured to be 3 s; therefore, the recycle delay was set to 3.9 s for 1D experiments. 256 scans (17 min) and 32 scans (2 min) were collected for the 1D spectra under microwave-off and microwave-on conditions, respectively. Without applying any window function that would broaden the spectra during processing, the DNP spectrum displayed linewidths approximated at a maximum of 2.8 ppm for partially resolved cellulose peaks. Spectral deconvolution was performed on the 95 to 30 ppm region using DMFit.^59^ The low chemical shift limit of the fit was chosen to show the baseline, while the higher cutoff was placed before the C1 signals. A minimum number of spectral components was chosen to fit the C4 region.

Two types of 2D ^13^C–^13^C correlation spectra were measured on the unlabeled CMF: a 2D refocused INADEQUATE spectrum that reports single quantum (SQ)–double quantum (DQ) correlations^60−62^ and a 2D CHHC spectrum that exhibits SQ–SQ correlations.^63^ The recycle delays were between 3.0 and 3.9 s. For CP-based refocused J-INADEQUATE, a total of 608 scans were recorded in 44 h over three repetitive experiments, with 74–80 points in the indirect dimension. For the CHHC spectrum, a total of 336 scans were recorded in 18 h over three repetitive experiments, with 26 to 58 points in the indirect dimension. The CP contact times for the first H–C CP, the second C–H CP, and the third H–C CP were 1000, 500, and 500 μs, respectively. A ^1^H–^1^H mixing period of 2 ms was used. The spectra presented here are the summations of all spectra for each experiment.

### Solid-State NMR of *Arabidopsis* Cell Walls

2D ^13^C–^13^C correlation solid-state NMR spectra were collected on uniformly ^13^C-labeled *Arabidopsis* samples for comparison with the DNP spectra collected on the unlabeled *in vitro* CMF. Isolation of the primary cell wall has been previously performed for intact and digested material.^64,65^ 1D ^13^C CP and 2D 30 ms proton-driven spin diffusion (PDSD) spectra were collected on both the digested and intact primary *Arabidopsis* cell walls on an 800 MHz NMR spectrometer under 13.5 kHz MAS frequency. The results were also compared with the spectra collected on a secondary *Arabidopsis* cell wall sample.^66^ A 2D CP refocused J-INADEQUATE spectrum of mature *Arabidopsis* stems (mainly secondary cell walls) was measured on a 600 MHz NMR under 14 kHz MAS at 293 K.

## Results and Discussion

### CET of *In Vitro* Fibers

**Figure 2:**
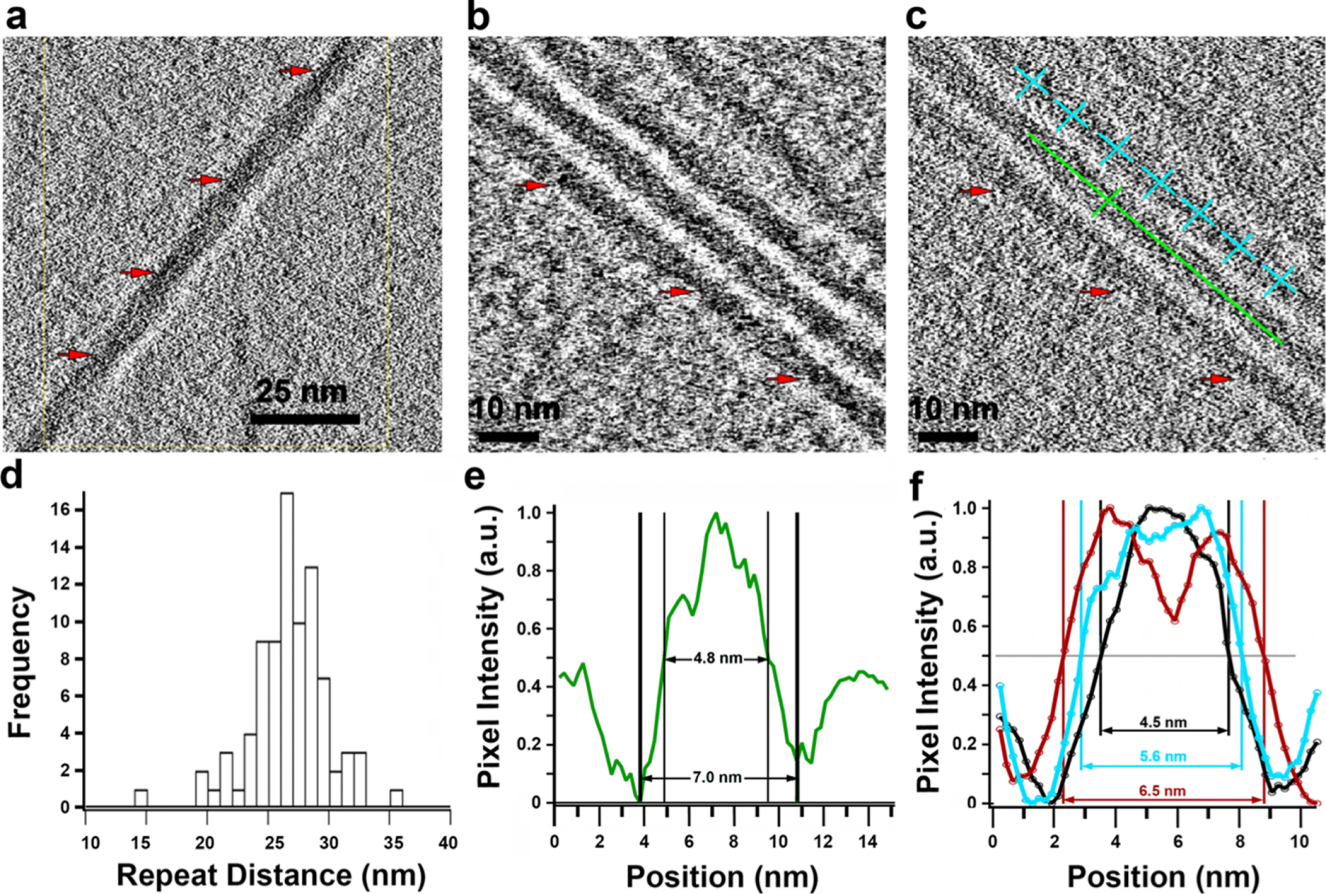
*In vitro* CMFs visualized as filaments in tomograms. (a) *In vitro* fibers with a periodic wrap or coil as visualized in a tomogram derived from a phase plate tilt series to enhance contrast (scale bar = 25 nm; red arrows point to a repeating darker, compressed region along the fiber). (b,c) Set of four fibers in a tomogram from a non-phase plate tilt series with contrast enhanced in (b) by averaging 25 slices along the *Z*-axis of the raw tomogram or not averaged in (c) (red arrows as in (a); scale bars = 10 nm). (d) Distribution of 72 repeat distances. (e) Pair of orthogonal green lines in (c) was used with the script “sideview-profile-average” to measure the diameter of the illustrated fiber (full width = 7.0 nm and FWHM = 4.8 nm). (f) Average of sideview-profile-averages for 100 pairs of orthogonal lines like the cyan ones in panel (c) that were placed at positions along several fibers randomly (cyan) at 50 of the darker repeats (black) or at 50 of the midpoints between the darker repeats (red). The FWHM fiber widths are shown in matching colors.

Within tomograms, *in vitro* fibers displayed a periodic repetition along their length (red arrows in Figure 2a–c). Most measurements of the repetition were within the range of 24–29 nm, with a mean periodicity of 26.7 ± 3.1 nm, as measured from the raw tomograms (Figure 2d). Fibers often ran parallel to one another, although isolated CMF were regularly seen. Parallel alignment may be attributed to the forces experienced during blotting of the grids immediately prior to plunge-freezing.

Using the script sideview-profile-average to generate a 1D profile of density across the fiber illustrated in Figure 2c (green lines), the diameter of a single *in vitro* fiber was 7.0 nm (full-width, FW approach; Figure 2e). Since it is difficult to deal with birefringence due to imaging at defocus, Nicolas *et al.*(46) used the FWHM method to measure microfiber diameter in cryo-electron tomograms of onion peels. For the single *in vitro* fiber analyzed in Figure 2e, the FWHM was 4.8 nm. In this measurement, the length of the long green guideline spanned more than one periodic repeat, so the density profile represents an average along the length of the fiber and does not reveal any possible variation in density along the fiber. Shorter guidelines (cyan lines in Figure 2c) were then used to measure profile densities at different segments along 100 fiber locations, the averages of which are shown in Figures 2f and S2a.

When placing the guidelines randomly along fibers, a broad profile was obtained (cyan), indicating a fiber diameter of 5.6 nm (FWHM). This profile was seen to be a sum of two distinct profiles when the guidelines were placed at 50 of the darker regions that were noted in Figure 2a–c (Figures 2f and S2b) or alternatively, at 50 of the midpoints between such darker regions (Figures 2f and S2c). The diameter of *in vitro* fibers thus varied periodically between 4.5 and 6.5 nm (FWHM), and the profile of the larger dimension could be modeled as two Gaussian peaks with FWHM diameters of 3.1 ± 0.1 and 2.7 ± 0.1 nm, respectively (Figure S2d). The smaller dimensions are close to the ∼3.5 nm width reported for CMF of plant cell walls measured by other methods^67^ and the larger dimension is nearly twice that size but very similar to the larger 6–10 nm subclasses of CMF observed by CET of cell walls present in *Arabidopsis* stems.^45^

**Figure 3:**
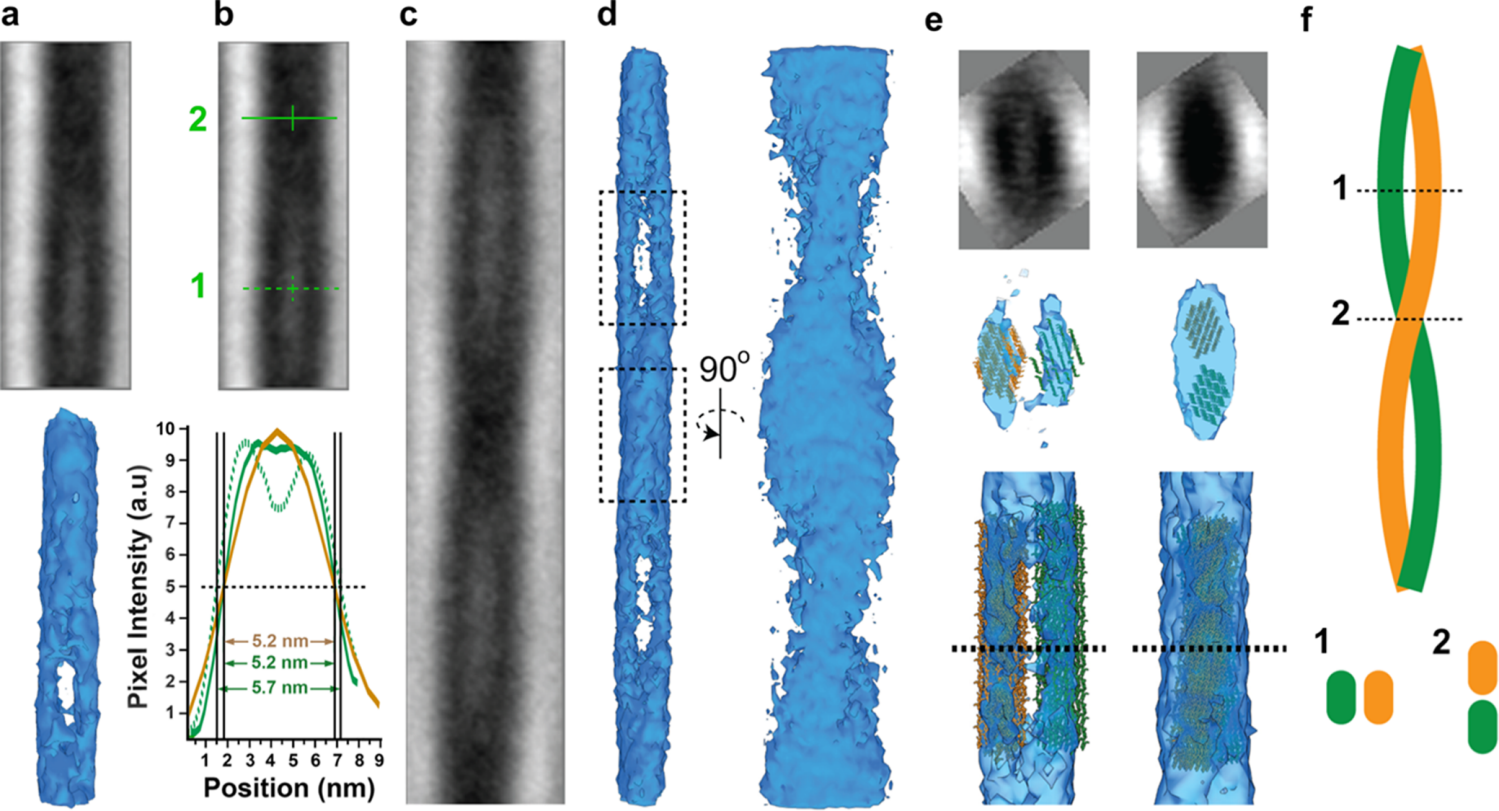
Subtomogram averages of *in vitro* fibers. (a) (Top) Slice through the subtomogram average of one periodic unit. (Bottom) Isosurface rendering of the density map shown above. (b) (Top) Slice through the subtomogram average as in (a) with regions marked (green, solid, and dashed) for sideview-profile-average analysis shown at the bottom, superimposed with a similar profile for untreated CMF of onion cell walls (gold).^46^ FWHM values for the three profiles are shown. (c) Slice through an expanded subtomogram average showing a full 360° wrap of the two sub-fibers. (d) Isosurface rendering of the density map in (c) from two different angles boxed regions enlarged in the bottom panel of (e). (e) (Top) Slices of the tomogram average showing cross-sections at the regions marked by the horizontal lines labeled 1 and 2 in (b). (Middle and Bottom) Two 18-chain cellulose models fit into the density map (d) by constrained rigid body fitting (orange and green represent two sub-fibers) showing the face-to-face arrangement of two 18-chain cellulose models (left) and the edge-to-edge arrangement (right). The middle panel shows the same cross-sections as the top panel, and the bottom panel shows an enlargement of the two regions boxed out in (d). (f) Schematic diagram of two wrapped filaments (orange and green) with locations of the cross-sections labeled 1 and 2. Note that the fibers, while wrapping, do not twist along the long axis.

To better explore the structure of *in vitro* fibers, we performed subtomogram averaging. Given the measured periodicity, model points were placed every 26.5 nm along the long axis of filaments in 12 different tomograms, and then these points were used to extract subtomograms. From all model points, 4377 subtomograms (52 × 126 × 52 pixels) were obtained, aligned to an initial reference, and then averaged and re-aligned iteratively to obtain a 25 Å resolution (FSC = 0.5) density map (Figure 3a). This average captured one apparent periodic unit of an *in vitro* CMF, which contained a pair of parallel fibers wrapping around one another.

To further characterize the repetitious nature of the CMF, a similar average was computed but with the particle size doubled along the long axis of the filament (52 × 256 × 52 pixels). Only every other model point was used so that the larger subtomograms did not overlap. This strategy gave half the particle count and a slightly reduced resolution (29.8 Å, FSC = 0.5), but it allowed us to view one complete 360° turn of the wrapping fibers (Figure 3c,d). At the center of this average, a crossover point between the fiber-pair is visible.

While wrapping, the electron density between fibers dropped over part of the trajectory and then increased over the rest of the run (as shown in Figure 3a and quantified by sideview-profile-averaging in Figure 3b). The FWHM values for the two regions were 5.2 and 5.7 nm, respectively. Analysis of the bimodal profile of the subtomogram average yielded two Gaussian peaks with FWHM values of 2.5 ± 0.1 and 3.3 ± 0.1 nm (Figure S2e), which are very similar to the values described above for individual fibers in the tomograms (Gaussian peaks of 2.7 and 3.1 nm, Figure S2d). In the larger subtomogram average, the two sub-filaments appeared to crossover edge-to-edge in the high-density regions and face-to-face in the low-density regions (as illustrated in Figure 3e,f, Movie S1, and Figure S3). We propose that the two sub-filaments helically wrap but do not twist around the long axis of their trajectory.

To our knowledge, the first CET of vitrified lamellae of plant cell walls was recently submitted for publication.^46^ The results of subtomogram averages of that study are directly comparable to those for the *in vitro* fibers being described here. FWHMs of the wrapped pair (5.2 and 5.7 nm at locations 1 and 2 in Figure 3b) fall near or within the 5.3 to 6.3 nm range of FWHMs reported for the onion CMF.^46^ In Figure 3b, the sideview-profile-average of CMFs in untreated onion walls is overlain with those of the *in vitro*-synthesized fibers. While of a very similar size, the *in vitro* fibers are not identical to the onion CMFs, as seen by the monomodal *versus* bimodal profiles depending on where along the *in vitro* fiber one looks. This feature could have been overlooked in the onion study, or could reflect structural differences due to *in vitro versus in vivo* synthesis conditions and the different resolutions achieved in the subtomogram averages. Using the FWHM measurements and taking each sub-filament of the *in vitro* fiber to be of equal size, each sub-filament is about 2.9 nm in diameter and shaped as expected for a modeled 18-chain crystalline cellulose microfiber, which we fit into the density map using constrained rigid body methods (Figure 3e). Together, the wrapped pair presents an oval cross-sectional area like the larger oval-shaped CMFs reported for *Arabidopsis* cell walls.^45^

Unfortunately, we have not obtained images of the *in vitro* CESA making glucan chains or of the chains coalescing into *in vitro* CMF. Likewise, the *in vivo* synthesis process has not been defined at such a level of detail. We thus do not know how faithfully the *in vitro* assembly process reflects the *in vivo* process. One potential difference is in the oligomerization state of the CESAs. So far, preparations of detergent-solubilized functional CESAs have yielded mixtures of monomers, dimers, and trimers, the latter giving rise to cryo-EM structures of putative CSC lobes,^6,68^ but higher-order assemblies like the hexamer of trimers seen in freeze-fracture TEM images of CSCs in plant cells have not been achieved *in vitro*. Also, higher-resolution structures are required to confirm or refute the possibility that the *in vitro*-synthesized fibers are crystalline cellulose. If they are, it is possible that the edge-to-edge fiber interactions seen here may contribute to the bundling of CMFs in plant cell walls. Below, we present DNP-assisted solid-state NMR data for the *in vitro* fibers that show them to be very similar to CMF in *Arabidopsis* cell walls but contain little bundling.

### DNP-Enabled Solid-State NMR Characterization of *In Vitro* Fibers

High-resolution structural characterization of the *in vitro*-synthesized CMF is technically challenging due to the lack of isotope-labeling and the low quantity of materials available for analysis (17 mg wet mass). Therefore, the enabling technique DNP is required to boost the NMR sensitivity by transferring polarization from the electrons to the nuclei.^69−74^ This will enable the use of the low natural abundance of ^13^C (1.1%) to measure multi-dimensional correlation spectra to probe the atomic-level structure of *in vitro* CMF. As summarized in Figure 1e,f, after *in vitro* synthesis by microsomal fraction enriched for CESA proteins,^26^ CMF is subjected to mixing with bi-radicals (AMUPol), the source of electrons for DNP, followed by DNP measurements on a 600 MHz/395 GHz instrument at cryogenic temperature.

**Figure 4:**
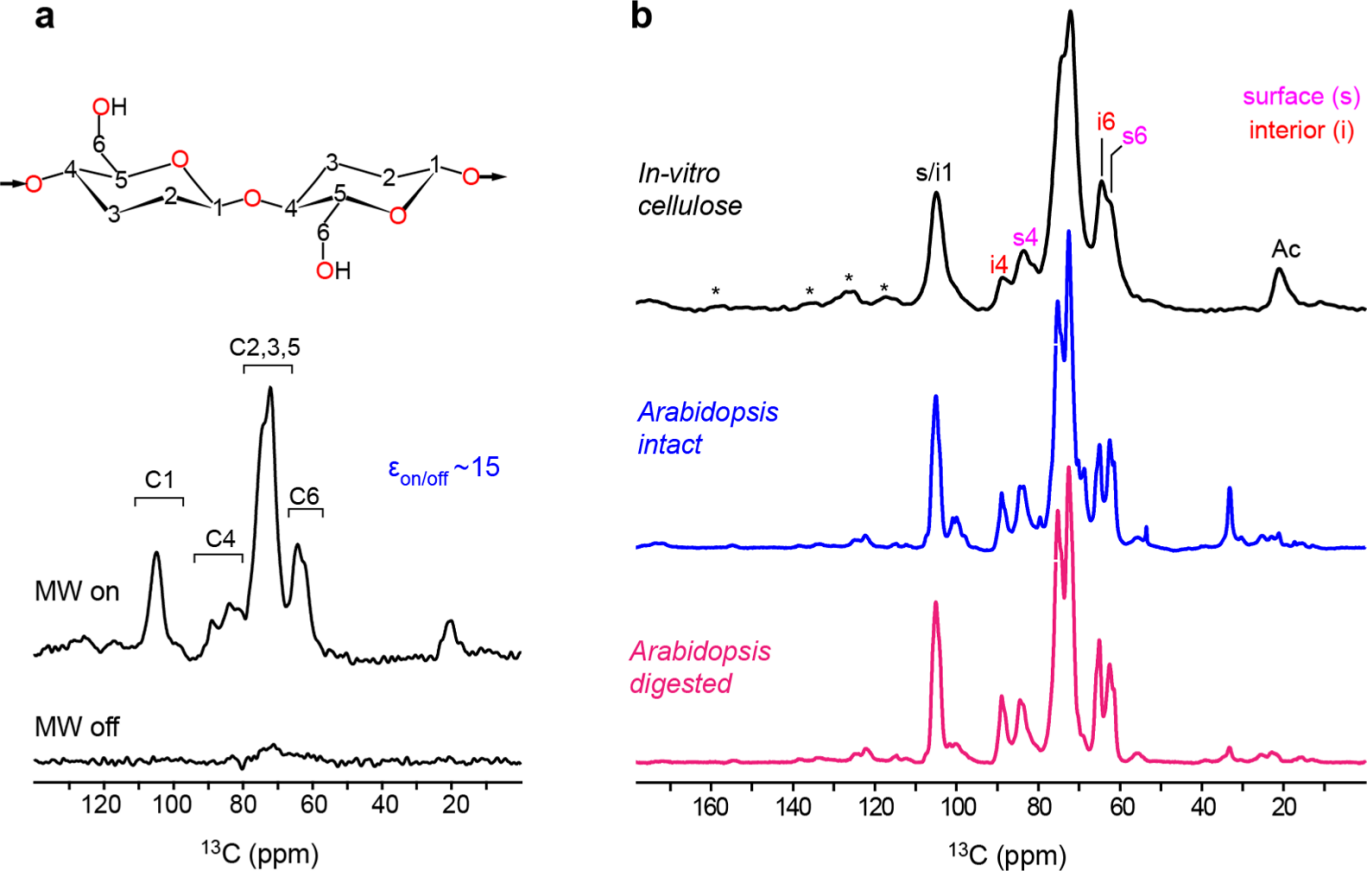
Structural analysis of *in vitro* CMFs enabled by the DNP method. (a) DNP spectra of *in vitro*-synthesized fibrils. The top panel shows the carbon numbering in the glucose units of cellulose. The bottom panel shows the comparison of ^13^C CP spectra (32 scans) with and without microwave (MW) irradiation. DNP enhances the signal-to-noise ratio by 15 times (ε_on/off_). (b) Spectral comparison of *in vitro* cellulose and *Arabidopsis* cell walls. From top to bottom are the ^13^C CP DNP spectrum of unlabeled CMF (256 scans) and the ^13^C CP NMR spectra of labeled *Arabidopsis* prior to and after enzymatic digestion of non-cellulosic components. The *in vitro* CMF spectrum was collected on a 600 MHz/395 GHz MAS-DNP instrument, and the *Arabidopsis* spectra was collected on an 800 MHz NMR. Stars (*) denote spinning sidebands, i and s, respectively, labeling interior and surface cellulose carbon assignments. Ac marks the cellulose acetate peak. Despite the temperature-induced line broadening, the spectrum features partially resolved interior and surface cellulose peaks.

The DNP technique is not detrimental to the analysis of biological cellulosic materials. Also, the spectral resolution of these highly crystalline microfibrils is largely retained at cryogenic temperature and after biradical addition as we have shown previously.^38,40,75^ Here we achieved a 15-fold increase in signal-to-noise as denoted by the enhancement factor ε_on/off_, which represents the ratio of peak intensities with and without microwave irradiation (Figure 4a). The pattern of the 1D ^13^C CP DNP spectrum generally followed that of the room-temperature spectra of ^13^C labeled cell walls of the model plant *Arabidopsis* (Figure 4b). Thus, the general spectral features of the interior and surface cellulose were resolved, notably in the C4 region with chemical shifts centered around 89 ppm for interior cellulose carbon-4 (i4) and 84 ppm for surface cellulose carbon-4 (s4). The other two domains of interest, according to their resolution, are the C1 peak at 105 ppm and the C6 signals at 65 and 62 ppm. As these chemical shifts are indicators of torsional conformations (*e.g.*, the χ torsion angle: O5–C5–C6–O6)^76^ and hydrogen-bonding patterns, the resemblance of spectral patterns has revealed the structural similarity of the glucose residues in the *in vitro* CMF and the plant cell wall CMFs.

In addition, the DNP spectrum also showed a carbonyl peak at 174 ppm and a methyl peak at 21 ppm, mutually assigned to the acetyl group of cellulose acetate.^77^ This derivative may be a consequence of enzymatic acetylation by the isolated protein apparatuses, which is a mixture of many membrane proteins contained by the detergent-solubilized microsomal fraction of protoplast membranes. Otherwise, it could be due to acetate formation in the short time lapse between DNP radical addition to the sample and its freezing.^78,79^ However, such a feature has never been observed in previous DNP samples of plant cell wall materials; it remains unclear if the *in vitro*-synthesized CMF has higher reactivity.

Cellulose relies on its crystallinity to maintain narrow NMR linewidths; therefore, cellulose signals are only moderately broadened by the cryogenic temperature during DNP experiments.^75^ In contrast, most non-cellulosic molecules, such as the matrix polysaccharides in plant cell walls, exhibit dramatically broadened signals at ∼100 K. For those dynamic molecules, a broad distribution of conformations will be entrapped (thus giving broad lines) when molecular motions are restricted under the DNP condition. The biradicals doped to the material preferentially partition into the solvent, using relayed ^1^H spin diffusion for hyperpolarization of molecules in the range of tens to hundreds of nanometers.^41^ The line-broadening effect by paramagnetic relaxation enhancement thus becomes minimal as assessed in multiple studies.^38,40,75^

**Figure 5:**
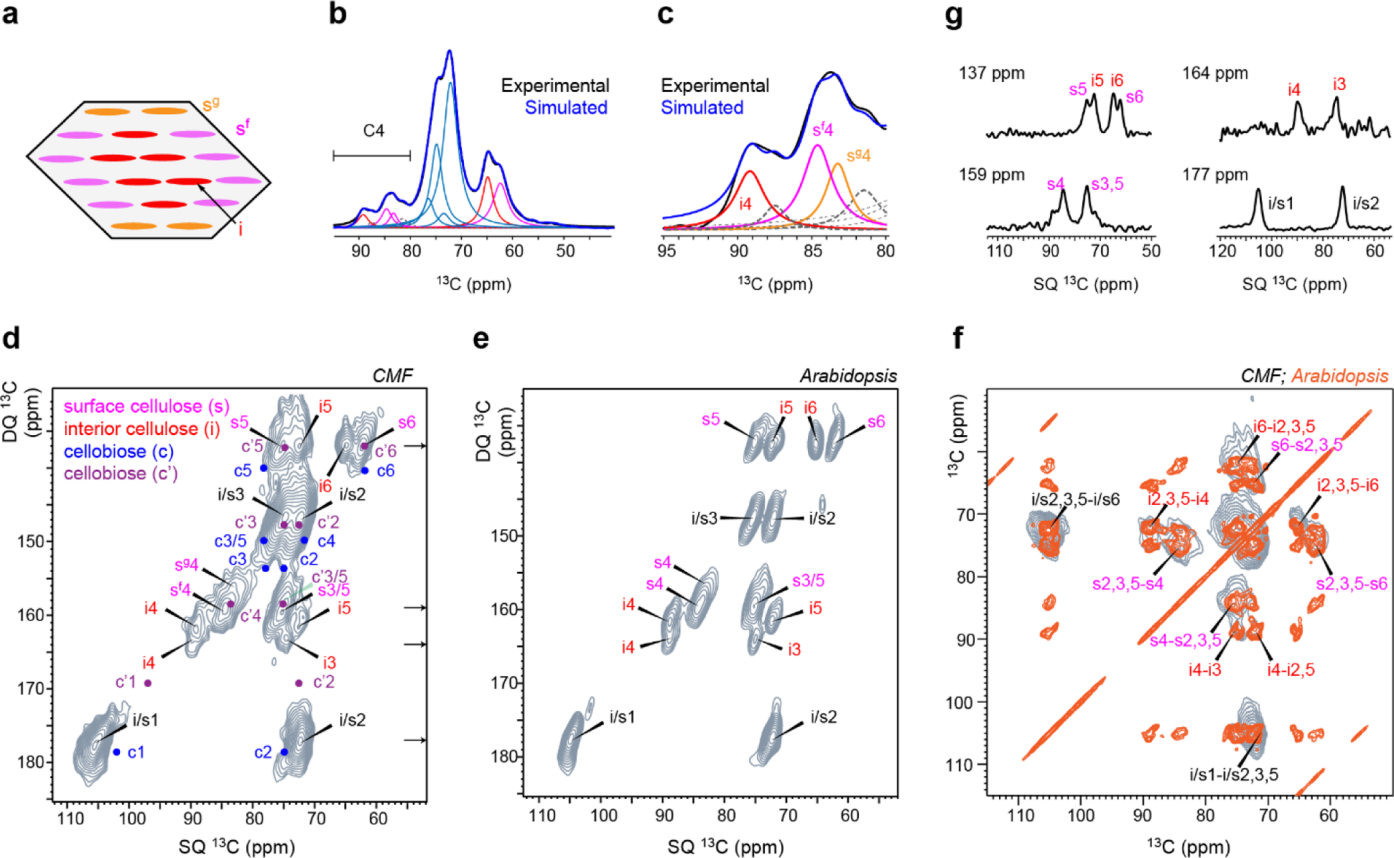
The structure of *in vitro* fibrils resembles that of plant cell wall cellulose. (a) Cross-section of a model fibril with 18 glucan chains, with one type of interior cellulose (i) and two surface units (s^f^ and s^g^). (b) Spectral deconvolution of CMF spectra in blue, matched to experimental data in black. (c) C4 region of the deconvolution. Major cellulose conformers are plotted in red, magenta, and orange, respectively, for types of interior cellulose and types (f,g) of surface cellulose. Thick dash lines correspond to two weak components in the i4 and s4 regions. Thin dash lines show the peak bases from C2,3,5 signals. (d) CP refocused the INADEQUATE spectrum of CMFs collected on a 600 MHz/395 GHz DNP. Surface cellulose spin pairs are assigned in magenta and interior cellulose in red. Expected cellobiose signals are transcribed in blue and purple. C1–C2 and C′1–C′2 pairs confirm no detection of cellobiose in CMF. (e) CP-based refocused INADEQUATE spectrum of secondary cell walls of *Arabidopsis* collected on a 600 MHz NMR. (f) Overlay of a tilted refocused INADEQUATE spectrum of CMF (gray) and a 30 ms PDSD spectrum of digested primary cell walls of *Arabidopsis* (orange) reveals an expected pattern of correlations in an SQ–SQ experiment. This *Arabidopsis* spectrum was collected on an 800 MHz NMR. (g) Assessment of the signal-to-noise ratios and linewidth from cross-sections sliced from panel (d).

While the number of glucan chains in cellulose has been under debate, mounting evidence from biochemical assays, imaging, modeling, and protein crystallography supports the concept that 18 chains should co-exist in an elementary microfibril.^6^ Density functional theory (DFT) calculations also suggest that each elementary microfibril might contain six layers of glucans in a 2-3-4-4-3-2 arrangement (Figure 5a).^80^ Solid-state NMR studies have recently revealed the torsional conformation of surface and interior chains (trans-gauche for interior chains and gauche-trans for surface chains) and have distinguished hydrophilic (s^f^) and hydrophobic (s^g^) surfaces.^76^ Spectral deconvolution was conducted using DMFit^59^ to analyze the composition of glucan chains, with a good agreement reached between the experimental and calculated spectra (Figure 5b; Tables S1 and S2). This fit was obtained while accounting for a major component at 89.2 ppm for interior cellulose but required two major peaks at 83.3 and 84.7 ppm for surface cellulose (Figure 5c). The complexity in data fitting indicates that *in vitro* CMF has generally retained the structural heterogeneity of cellulose in plants.

Two weak components were also identified in the deconvoluted spectrum (Figure 5c). The 87.6 ppm signal has a similar chemical shift to the type-c cellulose recently identified in intact plant cell walls.^34−36^ In plants, this special conformer belongs to some glucan chains that are deeply embedded in the core of a fibril, thus becoming spatially separated from surface chains. These chains cannot be accommodated by a small 18-chain microfibril; therefore, they might be created during the microfibril bundling process, which produces larger fibrils. The weak component of surface cellulose (81.5 ppm) was not well understood. A possible origin would be the presence of some more amorphous or less organized chains residing on the microfibril surface.

For better resolution, 2D correlation spectra were acquired on the unlabeled CMF, as enabled by the DNP technique. The refocused INADEQUATE spectrum collected on unlabeled CMFs (Figure 5d) and ^13^C-labeled *Arabidopsis* cell walls (Figure 5e) were highly comparable. This cell wall sample also has signals from non-cellulosic molecules such as xylan. The ^13^C chemical shifts of all resolved carbon sites in CMF have been summarized in Table S3. The structural similarity is further supported by the overlay of the tilted version of the refocused INADEQUATE spectrum of *in vitro* CMF with the 2D ^13^C–^13^C correlation spectrum of *Arabidopsis* cell walls (Figure 5f). Moreover, conformer-specific information was obtained from the C4 region, where positions of type-f and type-g surface cellulose C4 can be distinguished (Figure 5d). Within a short measurement time of 44 h, the 2D spectrum of unlabeled CMF has provided excellent resolution and sensitivity as evidenced by the extracted cross-sections (Figure 5g). The representative ^13^C FWHM linewidth is 1.8 ppm, with reasonably strong signals that are far beyond the noise level.

As the sample preparation procedures involved the mixing of CMF with cellobiose, a disaccharide formed by two glucose units, we need to determine if cellobiose has contributed to the signals of 2D spectra. The expected signals of the two glucose units of cellobiose^81^ deviate from the observed spectra, especially in the C1–C2 regions (Figure 5d). Cellulose has a high degree of polymerization through the C1–O–C4 covalent linkages. In contrast, the C1 of C′ glucose residue in cellobiose is not covalently linked to other sugar units, resulting in a unique C1 ^13^C chemical shift at 96 ppm. The signals of cellobiose have been broadened out by the broad distribution of conformations trapped at a low temperature; therefore, the DNP method selectively probes the highly crystalline component (CMF) in the sample. In addition, it is noteworthy that the expected chemical shifts of the model structures Iα and Iβ allomorphs^37^ do not match the measured spectra (Figure S4). This observation has further confirmed our previous findings that the model crystallographic structures cannot exist in cellulose fibers with small crystallite dimensions. The cellulose in most plant cell walls, as well as the *in vitro* CMF, does not follow the model structures characterized by diffraction methods.

Spectral integration of resolved peaks in the 2D refocused INADEQUATE spectrum (Table S4) and deconvolution of the 1D ^13^C CP spectrum (Table S1) are simultaneously conducted to quantify two structural aspects of *in vitro* CMF: the interior-to-surface ratio and the ratio of hydrophilic (type-f) and hydrophobic (type-g) surfaces. These two ratios shed light on the structure of CMF. The percentages of different glucan chains estimated from 1D and 2D experimental data are generally consistent with the numerical values predicted by the initial 18-chain model of CMFs (Table 1). The results indicate that the *in vitro* CMFs are mainly present as individual microfibrils instead of larger bundles.

**Table 1:** Distribution of Glucan Chains in *In Vitro* CMF^a^

glucan type in cellulose	percentage from model (%)	peak volume, 2D spectra (%)	peak area (i4, s4) 1D deconv.^b^ (%)	peak area (i4, s4) 1D deconv.^c^ (%)	peak area (i4, s4) 1D deconv.^d^ (%)	peak area (i6, s6) 1D deconv.
interior (i)	34	38	29	35	29	46%
surface (s)	66	62	71	65	71	54%
hydrophilic surface (s^f^)	66	60	60	60	60	NA
hydrophobic surface (s^g^)	34	40	40	40	40	NA

a Interior-to-surface ratios of CMF and the percentages of different surface conformers yielded from the theoretical model (from Figure 5a), peak volumes of 2D spectrum (from Figure 5d), and peak area of deconvoluted lines (from Figure 5c). Note that the i and s add up to 100% (all glucan chains are in a CMF). The s^f^ and s^g^ add up to 100% (all surface chain possible conformers). For 1D deconvolution, only resolved C4 and C6 signals are used. For 2D spectral analysis, all resolved resonances listed in Table S4 are used. NA: not available due to limited resolution.

b Peaks used for calculation: i4 (89.2 ppm), s^f^4 (84.7 ppm), and s^g^4 (83.3 ppm).

c Peaks: i4 (89.2 ppm), a minor i4 peak (87.6 ppm), s^f^4 (84.7 ppm), and s^g^4 (83.3 ppm).

d Peaks: i4 (89.2 ppm), a minor i4 peak (87.6 ppm), s^f^4 (84.7 ppm), s^g^4 (83.3 ppm), and a minor s4 peak (81.5 ppm).

The NMR analysis has a considerable error margin that cannot be avoided. This is because of the limited resolution in 1D spectra and the non-quantitative nature of 2D NMR (notably from the differences in *T*_2_ relaxation time constants for many carbon sites). For analysis based on peak volumes from 2D spectra, we have averaged all the resolved resonances (detailed in Table S4) to reduce the uncertainty. For analysis based on the area of deconvoluted C4 peaks in the 1D spectrum, three different ways were used to understand the error margin (Table 1). First, the interior cellulose content was estimated to be 29% if only the three major C4 peaks (i4 at 89.2 ppm, s^f^4 at 84.7 ppm, and s^g^4 at 83.3 ppm) were used for the calculation. Second, including the contribution of the weak peak at 87.6 ppm increased the content of interior cellulose to 35%. This minor component probably correlates with the type-c cellulose in plant cell walls, which belongs to a special form of glucan chains deeply embedded in the center of a bundle of microfibrils.^34,36^ The increase in the surface-to-interior ratio might reflect the structural effect of the bundling of microfibrils. Third, including the area of the 81.5 ppm peak (likely from some highly disordered surface chains) in the calculation will bring down the percentage of interior cellulose back to 29%. While these estimations based on C4 peak intensities gave a relatively good match to the model, the analysis based on C6 peaks gave a poor correlation, which is likely caused by the limited resolution of C6 signals.

**Figure 6:**
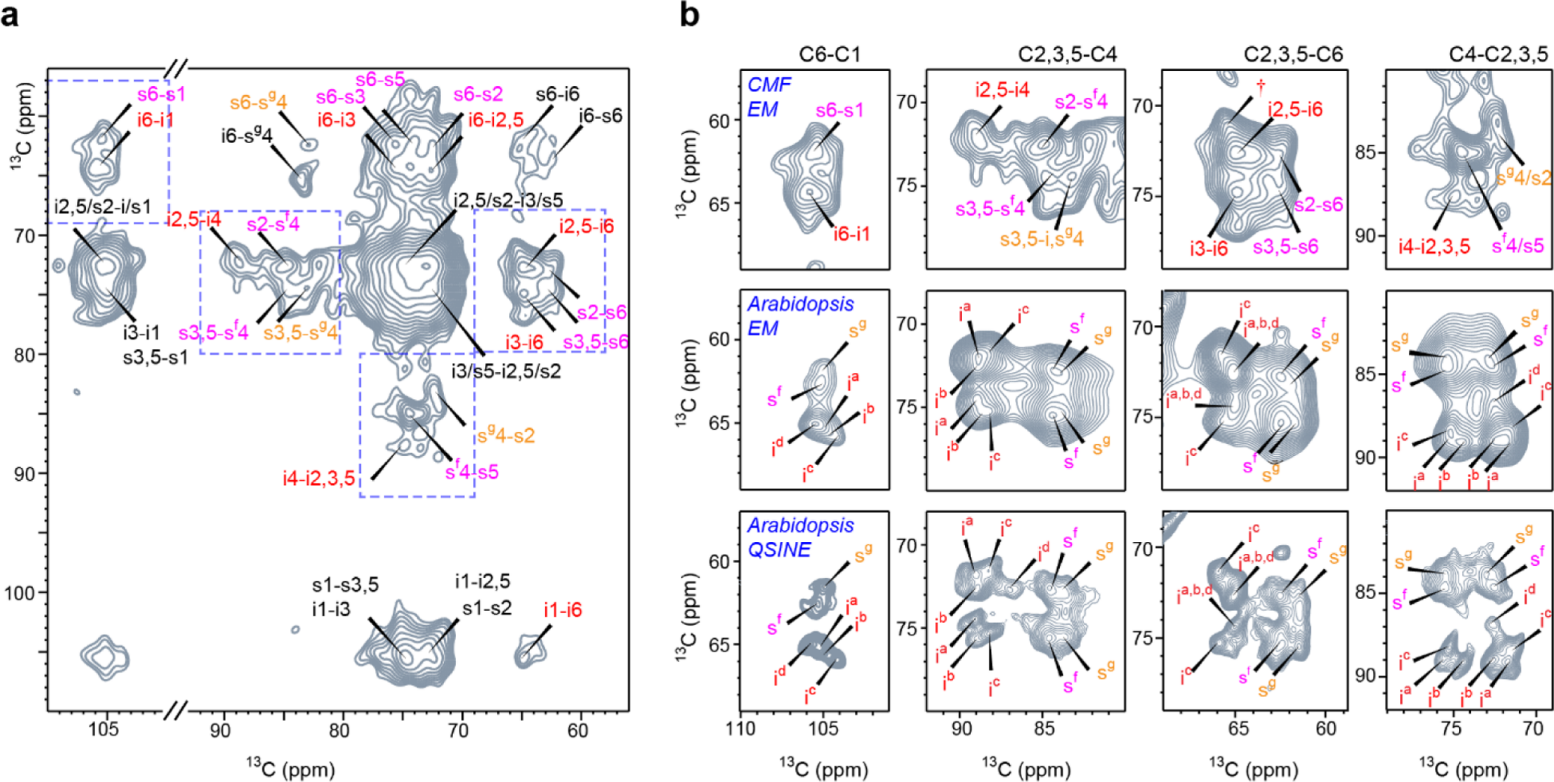
2D ^13^C–^13^C CHHC spectra reveal the conformational distribution of glucose units. (a) 2 ms CHHC correlation spectrum performed on CMF with off-diagonal resolved resonances assignment of interior cellulose in red, type-f surface cellulose in magenta and type-g unit in orange. Unresolved resonances are labeled in black. Blue dash lines mark zones of focus in the next panel. (b) Comparison of the *in vitro* CMF fiber and *Arabidopsis* cell wall cellulose. In the upper row, the same CHHC spectrum presented in panel (a) is re-plotted with a lowered baseline and additional contour levels to better view the conformer-specific signals. The middle row plotted the 30 ms PDSD spectrum of ^13^C-labeled digested *Arabidopsis* primary cell walls processed with the same window function as applied for CMF: exponential multiplication (EM) with a line broadening parameter of 100 Hz for both direct and indirect dimensions. The bottom row is the same *Arabidopsis* spectrum but processed with a squared sine bell (QSINE) window function with a Bruker TopSpin SSB parameter of 2.4. In the panel of C2,3,5–C6, † symbol marks possible signals of the type-c conformer of interior cellulose.

Finally, we attempt to further probe the CMF structure by acquiring an SQ–SQ correlation spectrum (Figure 6a). Under the natural abundance of ^13^C, most 2D SQ–SQ correlation methods are not functional: the spectra will be dominated by the diagonal as it is almost improbable for a ^13^C to correlate with another ^13^C to generate off-diagonal cross-peaks. However, the CHHC experiment chosen here will sufficiently suppress the diagonal due to the ^13^C–^1^H–^1^H–^13^C transfer pathway.^82^ This experimental scheme describes spatial correlations. Therefore, its sensitivity is substantially worse than the refocused INADEQUATE spectrum that only shows through-bond correlations. In total, 18 h of measurement are needed to obtain a satisfactory signal-to-noise ratio for the CHHC spectrum. The CHHC spectrum reports 18 one-bond cross-peaks (*e.g.*, s4–s5) and 28 multi-bond cross-peaks (*e.g.*, i1–i6) (Table S5). All cross-peaks involving interior and surface cellulose are well-resolved. In addition, three inter-glucan cross-peaks were observed, which happened between the interior chain carbon 6 and the carbon 4 of hydrophobic surface chains (i6-s^g^4) and between the carbon-6 sites of the internal and surface chains (s6–i6 and i6–s6).

A few spectral regions of CMF were compared with the 2D ^13^C–^13^C correlation spectra of ^13^C-labeled *Arabidopsis*-digested primary cell walls (Figure 6b). The *Arabidopsis* spectrum was presented with two types of window function processing: one with a squared sine bell (QSINE) window function that enhances resolution, and one with 100 Hz exponential (EM) broadening that partially enhances the signal-to-noise ratio and mimics CMF spectra. The *Arabidopsis* spectra showed the signals of different interior cellulose conformers, mainly type-c and type-a/b, which, respectively, correspond to the deeply embedded core chains and those intermediate layers sandwiched between the core and surface chains as we have resolved in previous studies.

With the current resolution, type-f and type-g surface conformers are easily differentiated for *in vitro* CMF, but this is not the case for interior cellulose conformers. Only one very weak peak shoulder could be partially observed in the C2,3,5–C6 region of the CMF CHHC spectrum. According to *Arabidopsis* data, this shoulder peak corresponds to a minor contribution of interior cellulose, c conformer, C6, which is present when the average structure of cellulose exceeds 18 chains, for example, through the association of multiple microfibrils. Its presence also explains why a minor additional component is necessary for 1D deconvolution (thick gray dashed line at 87.6 ppm in Figure 5c). As cellulose is rich in hydroxyl groups, chain bundling could be expected under the mediation of electrostatic interactions. The low intensity indicates that only a very low degree of bundling has occurred between different CMFs, which could occur either between very few chains fully parallel or between limited regions dispersed along the fibrils. This latter statement agrees with the tomography observation of the wrapping arrangement of CMF, leading to mostly individualized fibers and localized areas with higher cellulose densities (Movie S1 and Figure S3). As the interfibrillar association and sliding in the bundled cellulose networks regulate cell wall mechanics,^83^ understanding such interactions could guide the development of *in vitro* biomaterials with tunable properties.

## Conclusions

This study has presented CET subtomogram averaging of *in vitro*-synthesized CMFs and a MAS-DNP solid-state NMR method for characterizing their atomic-level structure without isotope-labeling and with a significantly limited quantity of material. DNP sensitivity enhancement has enabled the measurements of high-resolution 2D ^13^C–^13^C correlation spectra to resolve different glucan chains and quantify their populations in *in vitro* CMFs. Although synthesized *in vitro*, these CMFs have effectively retained the native structure of CMFs in plant cell walls. Quantification of peak intensities is in good agreement with the 18-chain cellulose model. Fibrillar bundling only occurs at a minimal level in *in vitro* CMFs, but there is an edge-to-edge interaction that might contribute to bundling. The methods are widely applicable to the structural elucidation of many other carbohydrate-based biomacromolecules such as functionalized cellulose- and lignocellulose-based fibers as well as *in vitro*-synthesized cell walls and biomaterials.

## Supporting Information Available

Comparison of three subtomogram averages; surface rendering of an 18-chain model of crystalline cellulose placed above the average for non-overlapping subtomograms of length 26.5 nm (left), 53.8 nm (middle), and 53.8 nm but with 50% overlap (right); scalebar is one average repeat length (26.7 nm) and below it is listed the density threshold for the current view; TEM of *in vitro*-synthesized fibrils; sideview-profile-averages of *in vitro* fibers and subtomogram average; snapshots of movie S1; overlay of CMF spectra with expected signals of Iα and Iβ cellulose; spectral deconvolution parameters; ^13^C chemical shifts of *in vitro* CMF; carbon–carbon correlations observed in the refocused INADEQUATE spectrum; integration intervals of the refocused INADEQUATE spectrum; and off-diagonal carbon–carbon correlations observed in the CHHC spectrum (PDF)
(MP4)

## Notes

The two subtomogram averages reported here have been submitted to the EMDB as EMD-25795 (10.9 × 26.5 × 10.9 nm, non-overlapping subtomograms) and EMD-25796 (10.9 × 53.8 × 10.9 nm, non-overlapping subtomograms).

## Abbreviations

CESA: cellulose synthase
CET: cryo-electron tomography
CMF: cellulose microfibrils
CP: cross-polarization
CSC: cellulose synthase complex
DFT: Density functional theory
DNP: dynamic nuclear polarization
DQ: double quantum
i: interior cellulose
INADEQUATE: incredible natural abundance double quantum transfer experiment
NMR: nuclear magnetic resonance
MAS: magic-angle spinning
PDSD: proton-driven spin diffusion
s: surface cellulose
SQ: single quantum
TEM: transmission electron microscope
TMS: tetramethylsilane
UDP-glucose: uridine diphosphate glucose

## References

[ref1] Cheah W. Y.; Sankaran R.; Show P. L.; Ibrahim T. N. B. T.; Chew K. W.; Culaba A.; Chang J.-S. Pretreatment methods for lignocellulosic biofuels production: current advances, challenges and future prospects. Biofuel Res. J. 2020, 7, 1115–1127. 10.18331/brj2020.7.1.4.

[ref2] Petridis L.; Smith J. C. Molecular-level driving forces in lignocellulosic biomass deconstruction for bioenergy. Nat. Rev. Chem. 2018, 2, 382–389. 10.1038/s41570-018-0050-6.

[ref3] Tu W.-C.; Hallett J. P. Recent advances in the pretreatment of lignocellulosic biomass. Curr. Opin. Green Sustain. Chem. 2019, 20, 11–17. 10.1016/j.cogsc.2019.07.004.

[ref4] Fatma S.; Hameed A.; Noman M.; Ahmed T.; Shahid M.; Tariq M.; Sohail I.; Tabassum R. Lignocellulosic Biomass: A Sustainable Bioenergy Source for the Future. Protein Pept. Lett. 2018, 25, 148–163. 10.2174/0929866525666180122144504. 29359659

[ref5] Lampugnani E. R.; Khan G. A.; Somssich M.; Persson S. Building a plant cell wall at a glance. J. Cell Sci. 2018, 131, jcs207373 10.1242/jcs.207373. 29378834

[ref6] Purushotham P.; Ho R.; Zimmer J. Architecture of a catalytically active homotrimeric plant cellulose synthase complex. Science 2020, 369, 1089–1094. 10.1126/science.abb2978. 32646917

[ref7] Jarvis M. Cellulose stacks up. Nature 2003, 426, 611–612. 10.1038/426611a. 14668842

[ref8] Fernandes A. N.; Thomas L. H.; Altaner C. M.; Callow P.; Forsyth V. T.; Apperley D. C.; Kennedy C. J.; Jarvis M. C. Nanostructure of cellulose microfibrils in spruce wood. Proc. Natl. Acad. Sci. U.S.A. 2011, 108, E1195–E1203. 10.1073/pnas.1108942108. 22065760 PMC3223458

[ref9] Zhong R.; Cui D.; Ye Z. H. Secondary cell wall biosynthesis. New Phytol. 2019, 221, 1703–1723. 10.1111/nph.15537. 30312479

[ref10] Verbančič J.; Lunn J. E.; Stitt M.; Persson S. Carbon Supply and the Regulation of Cell Wall Synthesis. Mol. Plant 2018, 11, 75–94. 10.1016/j.molp.2017.10.004. 29054565

[ref11] Somerville C. Cellulose Synthesis in Higher Plants. Annu. Rev. Cell Dev. Biol. 2006, 22, 53–78. 10.1146/annurev.cellbio.22.022206.160206. 16824006

[ref12] Thomas L. H.; Forsyth V. T.; Sturcová AA.; Kennedy C. J.; May R. P.; Altaner C.; Apperley D. C.; Wess T. J.; Jarvis M. C. Structure of Cellulose Microfibrils in Primary Cell Walls from Collenchyma. Plant Physiol. 2013, 161, 465–476. 10.1104/pp.112.206359. 23175754 PMC3532275

[ref13] Newman R. H.; Hill S. J.; Harris P. J. Wide-Angle X-Ray Scattering and Solid-State Nuclear Magnetic Resonance Data Combined to Test Models for Cellulose Microfibrils in Mung Bean Cell Walls. Plant Physiol. 2013, 163, 1558–1567. 10.1104/pp.113.228262. 24154621 PMC3846134

[ref14] Wang T.; Hong M. Solid-State NMR Investigations of Cellulose Structure and Interactions with Matrix Polysaccharides in Plant Primary Cell Walls. J. Exp. Bot. 2016, 67, 503–514. 10.1093/jxb/erv416. 26355148 PMC6280985

[ref15] Hill J. L.; Hammudi M. B.; Tien M. The Arabidopsis Cellulose Synthase Complex: A Proposed Hexamer of CESA Trimers in an Equimolar Stoichiometry. Plant Cell 2014, 26, 4834–4842. 10.1105/tpc.114.131193. 25490917 PMC4311198

[ref16] Juraniec M.; Gajda B. Cellulose biosynthesis in plants - the concerted action of CESA and non-CESA proteins. Biol. Plant. 2020, 64, 363–377. 10.32615/bp.2020.065.

[ref17] Li X.; Speicher T. L.; Dees D. C. T.; Mansoori N.; McManus J. B.; Tien M.; Trindade L. M.; Wallace I. S.; Roberts A. W. Convergent evolution of hetero-oligomeric cellulose synthesis complexes in mosses and seed plants. Plant J. 2019, 99, 862–876. 10.1111/tpj.14366. 31021018 PMC6711812

[ref18] Nixon B. T.; Mansouri K.; Singh A.; Du J.; Davis J. K.; Lee J. G.; Slabaugh E.; Vandavasi V. G.; O’Neill H.; Roberts E. M.; Roberts A. W.; Yingling Y. G.; Haigler C. H. Comparative Structural and Computational Analysis Supports Eighteen Cellulose Synthases in the Plant Cellulose Synthesis Complex. Sci. Rep. 2016, 6, 28696 10.1038/srep28696. 27345599 PMC4921827

[ref19] Vandavasi V. G.; Putnam D. K.; Zhang Q.; Petridis L.; Heller W. T.; Nixon B. T.; Haigler C. H.; Kalluri U.; Coates L.; Langan P.; Smith J. C.; Meiler J.; O’Neill H. A Structural Study of CESA1 Catalytic Domain of Arabidopsis Cellulose Synthesis Complex: Evidence for CESA Trimers. Plant Physiol. 2016, 170, 123–135. 10.1104/pp.15.01356. 26556795 PMC4704586

[ref20] Haigler C. H.; Grimson M. J.; Gervais J.; Le Moigne N.; Hofte H.; Monasse B.; Navard P. Molecular Modeling and Imaging of Initial Stages of Cellulose Fibril Assembly: Evidence for a Disordered Intermediate Stage. PLoS One 2014, 9, e93981 10.1371/journal.pone.0093981. 24722535 PMC3983097

[ref21] Polko J. K.; Kieber J. J. The Regulation of Cellulose Biosynthesis in Plants. Plant Cell 2019, 31, 282–296. 10.1105/tpc.18.00760. 30647077 PMC6447023

[ref22] Lai-Kee-Him J.; Chanzy H.; Müller M.; Putaux J.-L.; Imai T.; Bulone V. In Vitro Versus in VivoCellulose Microfibrils from Plant Primary Wall Synthases: Structural Differences. J. Biol. Chem. 2002, 277, 36931–36939. 10.1074/jbc.m203530200. 12145282

[ref23] Kudlicka K.; Brown R. M. Jr. Cellulose and Callose Biosynthesis in Higher Plants (I. Solubilization and Separation of (1->3)- and (1->4)-β-Glucan Synthase Activities from Mung Bean). Plant Physiol. 1997, 115, 643–656. 10.1104/pp.115.2.643. 12223833 PMC158525

[ref24] Colombani A.; Djerbi S.; Bessueille L.; Blomqvist K.; Ohlsson A.; Berglund T.; Teeri T. T.; Bulone V. In vitro synthesis of (1-3)-β-D-glucan (callose) and cellulose by detergent extracts of membranes from cell suspension cultures of hybrid aspen. Cellulose 2004, 11, 313–327. 10.1023/b:cell.0000046404.25406.19.

[ref25] Cifuentes C.; Bulone V.; Emons A. M. C. Biosynthesis of Callose and Cellulose by Detergent Extracts of Tobacco Cell Membranes and Quantification of the Polymers Synthesizedin vitro. J. Integr. Plant Biol. 2010, 52, 221–233. 10.1111/j.1744-7909.2010.00919.x. 20377683

[ref26] Cho S. H.; Du J.; Sines I.; Poosarla V. G.; Vepachedu V.; Kafle K.; Park Y. B.; Kim S. H.; Kumar M.; Nixon B. T. In vitro synthesis of cellulose microfibrils by a membrane protein from protoplasts of the non-vascular plant Physcomitrella patens. Biochem. J. 2015, 470, 195–205. 10.1042/bj20141391. 26348908

[ref27] Cho S. H.; Purushotham P.; Fang C.; Maranas C.; Díaz-Moreno S. M.; Bulone V.; Zimmer J.; Kumar M.; Nixon B. T. Synthesis and self-assembly of cellulose microfibrils from reconstituted cellulose synthase. Plant Physiol. 2017, 175, 146–156. 10.1104/pp.17.00619. 28768815 PMC5580757

[ref28] Purushotham P.; Cho S. H.; Díaz-Moreno S. M.; Kumar M.; Nixon B. T.; Bulone V.; Zimmer J. A single heterologously expressed plant cellulose synthase isoform is sufficient for cellulose microfibril formation in vitro. Proc. Natl. Acad. Sci. U.S.A. 2016, 113, 11360–11365. 10.1073/pnas.1606210113. 27647898 PMC5056052

[ref29] Foston M. Advances in solid-state NMR of cellulose. Curr. Opin. Biotechnol. 2014, 27, 176–184. 10.1016/j.copbio.2014.02.002. 24590189

[ref30] Ghassemi N.; Poulhazan A.; Deligey F.; Mentink-Vigier F.; Marcotte I.; Wang T. Solid-State NMR Investigations of Extracellular Matrixes and Cell Walls of Algae, Bacteria, Fungi, and Plants. Chem. Rev. 2021, in press. 10.1021/acs.chemrev.1c00669 PMC9486976 34878762

[ref31] Zhao W.; Fernando L. D.; Kirui A.; Deligey F.; Wang T. Solid-state NMR of plant and fungal cell walls: a critical review. Solid State Nucl. Magn. Reson. 2020, 107, 101660 10.1016/j.ssnmr.2020.101660. 32251983

[ref32] El Hariri El Nokab M.; Van der Wel P. C. A. Use of solid-state NMR spectroscopy for investigating polysaccharide-based hydrogels: A review. Carbohydr. Polym. 2020, 240, 116276 10.1016/j.carbpol.2020.116276. 32475563

[ref33] Giummarella N.; Pu Y.; Ragauskas A. J.; Lawoko M. A critical review on the analysis of lignin carbohydrate bonds. Green Chem. 2019, 21, 1573–1595. 10.1039/c8gc03606c.

[ref34] Wang T.; Yang H.; Kubicki J. D.; Hong M. Cellulose Structural Polymorphism in Plant Primary Cell Walls Investigated by High-Field 2D Solid-State NMR Spectroscopy and Density Functional Theory Calculations. Biomacromolecules 2016, 17, 2210–2222. 10.1021/acs.biomac.6b00441. 27192562 PMC5270591

[ref35] Kang X.; Kirui A.; Dickwella Widanage M. C.; Mentink-Vigier F.; Cosgrove D. J.; Wang T. Lignin-polysaccharide interactions in plant secondary cell walls revealed by solid-state NMR. Nat. Commun. 2019, 10, 347 10.1038/s41467-018-08252-0. 30664653 PMC6341099

[ref36] Kirui A.; Zhao W.; Deligey F.; Yang H.; Kang X.; Mentink-Vigier F.; Wang T. Carbohydrate-aromatic interface and molecular architecture of lignocellulose. Nat. Commun. 2022, 13, 538 10.1038/s41467-022-28165-3. 35087039 PMC8795156

[ref37] Kono H.; Numata Y. Structural investigation of cellulose Iα and Iβ by 2D RFDR NMR spectroscopy: determination of sequence of magnetically inequivalent d-glucose units along cellulose chain. Cellulose 2006, 13, 317–326. 10.1007/s10570-005-9025-0.

[ref38] Kirui A.; Ling Z.; Kang X.; Dickwella Widanage M. C.; Mentink-Vigier F.; French A. D.; Wang T. Atomic resolution of cotton cellulose structure enabled by dynamic nuclear polarization solid-state NMR. Cellulose 2019, 26, 329–339. 10.1007/s10570-018-2095-6. 31289425 PMC6615758

[ref39] Chakraborty A.; Deligey F.; Quach J.; Mentink-Vigier F.; Wang P.; Wang T. Biomolecular complex viewed by dynamic nuclear polarization solid-state NMR spectroscopy. Biochem. Soc. Trans. 2020, 48, 1089–1099. 10.1042/bst20191084. 32379300 PMC7565284

[ref40] Zhao W.; Kirui A.; Deligey F.; Mentink-Vigier F.; Zhou Y.; Zhang B.; Wang T. Solid-state NMR of unlabeled plant cell walls: high-resolution structural analysis without isotopic enrichment. Biotechnol. Biofuels 2021, 14, 14 10.1186/s13068-020-01858-x. 33413580 PMC7792314

[ref41] Viger-Gravel J.; Lan W.; Pinon A. C.; Berruyer P.; Emsley L.; Bardet M.; Luterbacher J. Topology of Pretreated Wood Fibers Using Dynamic Nuclear Polarization. J. Phys. Chem. C 2019, 123, 30407–30415. 10.1021/acs.jpcc.9b09272.

[ref42] Perras F. A.; Luo H.; Zhang X.; Mosier N. S.; Pruski M.; Abu-Omar M. M. Atomic-Level Structure Characterization of Biomass Pre- and Post-Lignin Treatment by Dynamic Nuclear Polarization-Enhanced Solid-State NMR. J. Phys. Chem. A 2017, 121, 623–630. 10.1021/acs.jpca.6b11121. 28026949

[ref43] Berruyer P.; Gericke M.; Moutzouri P.; Jakobi D.; Bardet M.; Karlson L.; Schantz S.; Heinze T.; Emsley L. Advanced characterization of regioselectively substituted methylcellulose model compounds by DNP enhanced solid-state NMR spectroscopy. Carbohydr. Polym. 2021, 262, 117944 10.1016/j.carbpol.2021.117944. 33838821

[ref44] Sarkar P.; Bosneaga E.; Yap E. G. Jr.; Das J.; Tsai W.-T.; Cabal A.; Neuhaus E.; Maji D.; Kumar S.; Joo M.; Yakovlev S.; Csencsits R.; Yu Z.; Bajaj C.; Downing K. H.; Auer M. Electron tomography of cryo-immobilized plant tissue: a novel approach to studying 3D macromolecular architecture of mature plant cell walls in situ. PLoS One 2014, 9, e106928 10.1371/journal.pone.0106928. 25207917 PMC4160213

[ref45] Sarkar P.; Kowalczyk M.; Apte S.; Yap E. G.; Das J.; Adams P. D.; Bajaj C.; Guindos P.; Auer M. Cryo-Electron Tomography 3D Structure and Nanoscale Model of Arabidopsis thaliana Cell Wall. Submission date: Dec 10, 2018, bioRxiv:492140. (accessed Feb 25, 2022).

[ref46] Nicolas W. J.; Fäßler F.; Dutka P.; Schur F. K. M.; Jensen G.; Meyerowitz E. Bimodally oriented cellulose fibers and reticulated homogalacturonan networks - A direct visualization of Allium cepa primary cell walls. Submission date: Feb 01, 2022, bioRxiv:478342 (accessed Feb 25, 2022).

[ref47] Makarem M.; Lee C. M.; Kafle K.; Huang S.; Chae I.; Yang H.; Kubicki J. D.; Kim S. H. Probing cellulose structures with vibrational spectroscopy. Cellulose 2019, 26, 35–79. 10.1007/s10570-018-2199-z.

[ref48] Menand B.; Calder G.; Dolan L. Both chloronemal and caulonemal cells expand by tip growth in the moss Physcomitrella patens. J. Exp. Bot. 2007, 58, 1843–1849. 10.1093/jxb/erm047. 17404383

[ref49] Bharat T. A. M.; Scheres S. H. W. Resolving macromolecular structures from electron cryo-tomography data using subtomogram averaging in RELION. Nat. Protoc. 2016, 11, 2054–2065. 10.1038/nprot.2016.124. 27685097 PMC5215819

[ref50] Mastronarde D. N. Automated electron microscope tomography using robust prediction of specimen movements. J. Struct. Biol. 2005, 152, 36–51. 10.1016/j.jsb.2005.07.007. 16182563

[ref51] Ortega D. R.; Yang W.; Subramanian P.; Mann P.; Kjær A.; Chen S.; Watts K. J.; Pirbadian S.; Collins D. A.; Kooger R.; Kalyuzhnaya M. G.; Ringgaard S.; Briegel A.; Jensen G. J. Repurposing a chemosensory macromolecular machine. Nat. Commun. 2020, 11, 2041 10.1038/s41467-020-15736-5. 32341341 PMC7184735

[ref52] Nicastro D.; Schwartz C.; Pierson J.; Gaudette R.; Porter M. E.; McIntosh J. R. The molecular architecture of axonemes revealed by cryoelectron tomography. Science 2006, 313, 944–948. 10.1126/science.1128618. 16917055

[ref53] Heumann J. M.; Hoenger A.; Mastronarde D. N. Clustering and variance maps for cryo-electron tomography using wedge-masked differences. J. Struct. Biol. 2011, 175, 288–299. 10.1016/j.jsb.2011.05.011. 21616153 PMC3150390

[ref54] Takahashi H.; Lee D.; Dubois L.; Bardet M.; Hediger S.; De Paëpe G. Rapid Natural-Abundance 2D ^13^C–^13^C Correlation Spectroscopy Using Dynamic Nuclear Polarization Enhanced Solid-State NMR and Matrix-Free Sample Preparation. Angew. Chem., Int. Ed. 2012, 51, 11766–11769. 10.1002/anie.201206102. 23081784

[ref55] Sauvée C.; Rosay M.; Casano G.; Aussenac F.; Weber R. T.; Ouari O.; Tordo P. Highly Efficient, Water-Soluble Polarizing Agents for Dynamic Nuclear Polarization at High Frequency. Angew. Chem., Int. Ed. 2013, 52, 10858–10861. 10.1002/anie.201304657. 23956072

[ref56] Akbey Ü.; Franks W. T.; Linden A.; Lange S.; Griffin R. G.; van Rossum B.-J.; Oschkinat H. Dynamic Nuclear Polarization of Deuterated Proteins. Angew. Chem., Int. Ed. 2010, 49, 7803–7806. 10.1002/anie.201002044. PMC4435681 20726023

[ref57] Kirui A.; Dickwella Widanage M. C.; Mentink-Vigier F.; Wang P.; Kang X.; Wang T. Preparation of Fungal and Plant Materials for Structural Elucidation Using Dynamic Nuclear Polarization Solid-State NMR. J. Visualized Exp. 2019, 144, e59152 10.3791/59152. 30829332

[ref58] Liao S. Y.; Lee M.; Wang T.; Sergeyev I. V.; Hong M. Efficient DNP NMR of membrane proteins: sample preparation protocols, sensitivity, and radical location. J. Biol. NMR 2016, 64, 223–237. 10.1007/s10858-016-0023-3. PMC4826309 26873390

[ref59] Massiot D.; Fayon F.; Capron M.; King I.; Le Calvé S.; Alonso B.; Durand J.-O.; Bujoli B.; Gan Z.; Hoatson G. Modelling one- and two-dimensional solid-state NMR spectra. Magn. Reson. Chem. 2002, 40, 70–76. 10.1002/mrc.984.

[ref60] Cadars S.; Sein J.; Duma L.; Lesage A.; Pham T. N.; Baltisberger J. H.; Brown S. P.; Emsley L. The refocused INADEQUATE MAS NMR experiment in multiple spin-systems: Interpreting observed correlation peaks and optimising lineshapes. J. Magn. Reson. 2007, 188, 24–34. 10.1016/j.jmr.2007.05.016. 17588789

[ref61] Lesage A.; Bardet M.; Emsley L. Through-Bond Carbon–Carbon Connectivities in Disordered Solids by NMR. J. Am. Chem. Soc. 1999, 121, 10987–10993. 10.1021/ja992272b.

[ref62] Fayon F.; Massiot D.; Levitt M. H.; Titman J. J.; Gregory D. H.; Duma L.; Emsley L.; Brown S. P. Through-space contributions to two-dimensional double-quantum J correlation NMR spectra of magic-angle-spinning solids. J. Chem. Phys. 2005, 122, 194313 10.1063/1.1898219. 16161579

[ref63] Aluas M.; Tripon C.; Griffin J. M.; Filip X.; Ladizhansky V.; Griffin R. G.; Brown S. P.; Filip C. CHHC and 1H-1H magnetization exchange: Analysis by experimental solid-state NMR and 11-spin density-matrix simulations. J. Magn. Reson. 2009, 199, 173–187. 10.1016/j.jmr.2009.04.013. 19467890 PMC2706310

[ref64] Wang T.; Park Y. B.; Cosgrove D. J.; Hong M. Cellulose-Pectin Spatial Contacts Are Inherent to Never-Dried Arabidopsis Primary Cell Walls: Evidence from Solid-State Nuclear Magnetic Resonance. Plant Physiol. 2015, 168, 871–884. 10.1104/pp.15.00665. 26036615 PMC4741345

[ref65] White P. B.; Wang T.; Park Y. B.; Cosgrove D. J.; Hong M. Water-Polysaccharide Interactions in the Primary Cell Wall of Arabidopsis thaliana from Polarization Transfer Solid-State NMR. J. Am. Chem. Soc. 2014, 136, 10399–10409. 10.1021/ja504108h. 24984197

[ref66] Dupree R.; Simmons T. J.; Mortimer J. C.; Patel D.; Iuga D.; Brown S. P.; Dupree P. Probing the Molecular Architecture of Arabidopsis thaliana Secondary Cell Walls Using Two- and Three-Dimensional 13C Solid State Nuclear Magnetic Resonance Spectroscopy. Biochemistry 2015, 54, 2335–2345. 10.1021/bi501552k. 25739924

[ref67] Zhang T.; Zheng Y.; Cosgrove D. J. Spatial organization of cellulose microfibrils and matrix polysaccharides in primary plant cell walls as imaged by multichannel atomic force microscopy. Plant J. 2016, 85, 179–192. 10.1111/tpj.13102. 26676644

[ref68] Zhang X.; Xue Y.; Guan Z.; Zhou C.; Nie Y.; Men S.; Wang Q.; Shen C.; Zhang D.; Jin S.; Tu L.; Yin P.; Zhang X. Structural insights into homotrimeric assembly of cellulose synthase CesA7 from Gossypium hirsutum. Plant Biotechnol. J. 2021, 19, 1579–1587. 10.1111/pbi.13571. 33638282 PMC8384604

[ref69] Mentink-Vigier F.; Akbey Ü.; Oschkinat H.; Vega S.; Feintuch A. Theoretical aspects of Magic Angle Spinning - Dynamic Nuclear Polarization. J. Magn. Reson. 2015, 258, 102–120. 10.1016/j.jmr.2015.07.001. 26232770

[ref70] Su Y.; Andreas L.; Griffin R. G. Magic Angle Spinning NMR of Proteins: High-Frequency Dynamic Nuclear Polarization and ^1^H Detection. Annu. Rev. Biochem. 2015, 84, 465–497. 10.1146/annurev-biochem-060614-034206. 25839340

[ref71] Rossini A. J.; Zagdoun A.; Lelli M.; Lesage A.; Copéret C.; Emsley L. Dynamic Nuclear Polarization Surface Enhanced NMR Spectroscopy. Acc. Chem. Res. 2013, 46, 1942–1951. 10.1021/ar300322x. 23517009

[ref72] Jaudzems K.; Polenova T.; Pintacuda G.; Oschkinat H.; Lesage A. DNP NMR of biomolecular assemblies. J. Struct. Biol. 2019, 206, 90–98. 10.1016/j.jsb.2018.09.011. 30273657

[ref73] Cheng C.-Y.; Han S. Dynamic Nuclear Polarization Methods in Solids and Solutions to Explore Membrane Proteins and Membrane Systems. Annu. Rev. Phys. Chem. 2013, 64, 507–532. 10.1146/annurev-physchem-040412-110028. 23331309

[ref74] Mandala V. S.; Hong M. High-sensitivity protein solid-state NMR spectroscopy. Curr. Opin. Struct. Biol. 2019, 58, 183–190. 10.1016/j.sbi.2019.03.027. 31031067 PMC6778492

[ref75] Wang T.; Park Y. B.; Caporini M. A.; Rosay M.; Zhong L.; Cosgrove D. J.; Hong M. Sensitivity-enhanced solid-state NMR detection of expansin’s target in plant cell walls. Proc. Natl. Acad. Sci. U.S.A. 2013, 110, 16444–16449. 10.1073/pnas.1316290110. 24065828 PMC3799313

[ref76] Phyo P.; Wang T.; Yang Y.; O’Neill H.; Hong M. Direct determination of hydroxymethyl conformations of plant cell wall cellulose using ^1^H polarization transfer solid-state NMR. Biomacromolecules 2018, 19, 1485–1497. 10.1021/acs.biomac.8b00039. 29562125

[ref77] Doyle S.; Pethrick R. A.; Harris R. K.; Lane J. M.; Packer K. J.; Heatley F. 13C nuclear magnetic resonance studies of cellulose acetate in the solution and solid states. Polymer 1986, 27, 19–24. 10.1016/0032-3861(86)90351-4.

[ref78] Gomez-Bujedo S.; Fleury E.; Vignon M. R. Preparation of cellouronic acids and partially acetylated cellouronic acids by TEMPO/NaClO oxidation of water-soluble cellulose acetate. Biomacromolecules 2004, 5, 565–571. 10.1021/bm034405y. 15003022

[ref79] Isogai A.; Kato Y. Preparation of polyglucuronic acid from cellulose by TEMPO-mediated oxidation. Cellulose 1998, 5, 153–164. 10.1023/a:1009208603673.

[ref80] Yang H.; Kubicki J. D. A density functional theory study on the shape of the primary cellulose microfibril in plants: effects of C6 exocyclic group conformation and H-bonding. Cellulose 2020, 27, 2389–2402. 10.1007/s10570-020-02970-9.

[ref81] Tang H. R.; Belton P. S. Molecular dynamics of polycrystalline cellobiose studied by solid-state NMR. Solid State Nucl. Magn. Reson. 2002, 21, 117–133. 10.1006/snmr.2002.0052. 12199355

[ref82] Kobayashi T.; Slowing I. I.; Pruski M. Measuring Long-Range ^13^C–^13^C Correlations on a Surface under Natural Abundance Using Dynamic Nuclear Polarization-Enhanced Solid-State Nuclear Magnetic Resonance. J. Phys. Chem. C 2017, 121, 24687–24691. 10.1021/acs.jpcc.7b08841.

[ref83] Zhang Y.; Yu J.; Wang X.; Durachko D. M.; Zhang S.; Cosgrove D. J. Molecular insights into the complex mechanics of plant epidermal cell walls. Science 2021, 372, 706–711. 10.1126/science.abf2824. 33986175

