# Supplementary material for: Structure of In Vitro-Synthesized Cellulose Fibrils Viewed by Cryo-Electron Tomography and 13C Natural-Abundance Dynamic Nuclear Polarization Solid-State NMR
Source: Biomacromolecules. 2022 Mar 26;23(6):2290–301. doi: 10.1021/acs.biomac.1c01674 (PMC9198983; doi:10.1021/acs.biomac.1c01674)
Supplement: Supplementary file 1 — bm1c01674_si_001.pdf [file bm1c01674_si_001.pdf]

Supporting Information

Structure of *In-Vitro* Synthesized Cellulose Fibrils Viewed by Cryo-Electron Tomography and ^{13}C Natural-Abundance Dynamic Nuclear Polarization Solid-State NMR

Fabien Deligey[1‡], Mark A. Frank[2‡], Sung Hyun Cho[2], Alex Kirui[1], Frederic Mentink-Vigier[3], Matthew T. Swulius[4], B. Tracy Nixon[2], and Tuo Wang[1*].*

[1] Department of Chemistry, Louisiana State University, Baton Rouge, LA 70803, USA

[2] Department of Biochemistry and Molecular Biology, Pennsylvania State University, University Park, PA 16802, USA

[3] National High Magnetic Field Laboratory, Tallahassee, FL 32310, USA

[4] Department of Biochemistry and Molecular Biology, Pennsylvania State University, Hershey, PA 17033, USA

[‡]These authors contributed equally

* Correspondence and requests for materials should be addressed to:

T.W. (email: tuowang@lsu.edu); B.T.N. (email: btn1@psu.edu)

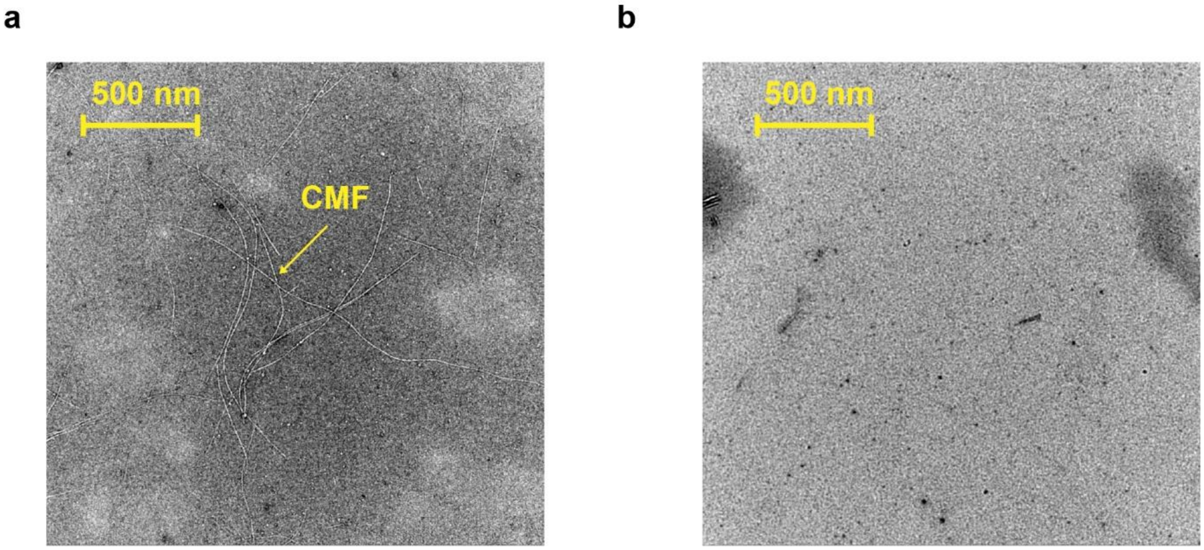

Figure S1. Fibril formation confirmed by TEM. (a) TEM of cellulose microfibrils synthesized by membrane proteins isolated from the moss *Physcomitrella patens* overexpressing HA-tagged PpCESA5, which were incubated with UDP-glucose. Arrow points to an individual fibril. **(b)** TEM of a control setup without the supply of UDP-glucose. No fibril is observed.

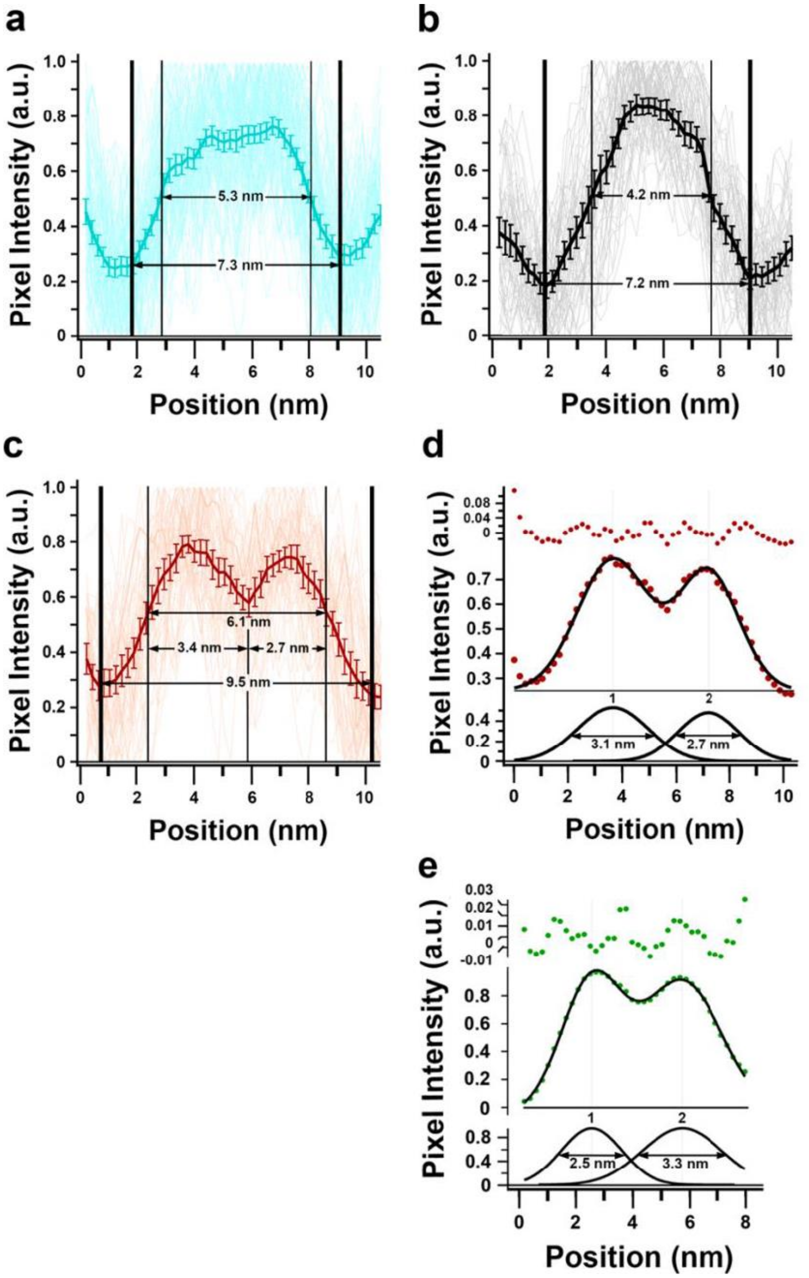

Figure S2. Sideview-profile-averages of *in-vitro* fibers and subtomogram average. (a) One-hundred profiles of segments of fibers are plotted for guidelines randomly positioned along fibers as illustrated in Figure 2c (cyan), with the average and 95% confidence interval enlarged and FW and FWHM distances drawn. **(b)** Same as in (a) but for 50 profiles with guidelines placed at the darker repeat elements as illustrated by the red arrows in Figure 2a-c. **(c)** Same as in (b) but for 50 profiles with guidelines placed at the midpoint between darker repeat elements. **(d)** Fit to two Gaussian peaks for average profile in (c); FWHMs of 2.7 ± 0.1 nm and 3.1 ± 0.1 nm are shown. **(e)** Fit to 2 Gaussian peaks for single profile at midpoint between crossover points of the subtomogram average in Figure 3a,b; FWHMs of 2.5 ± 0.1 nm and 3.3 ± 0.1 nm are shown.

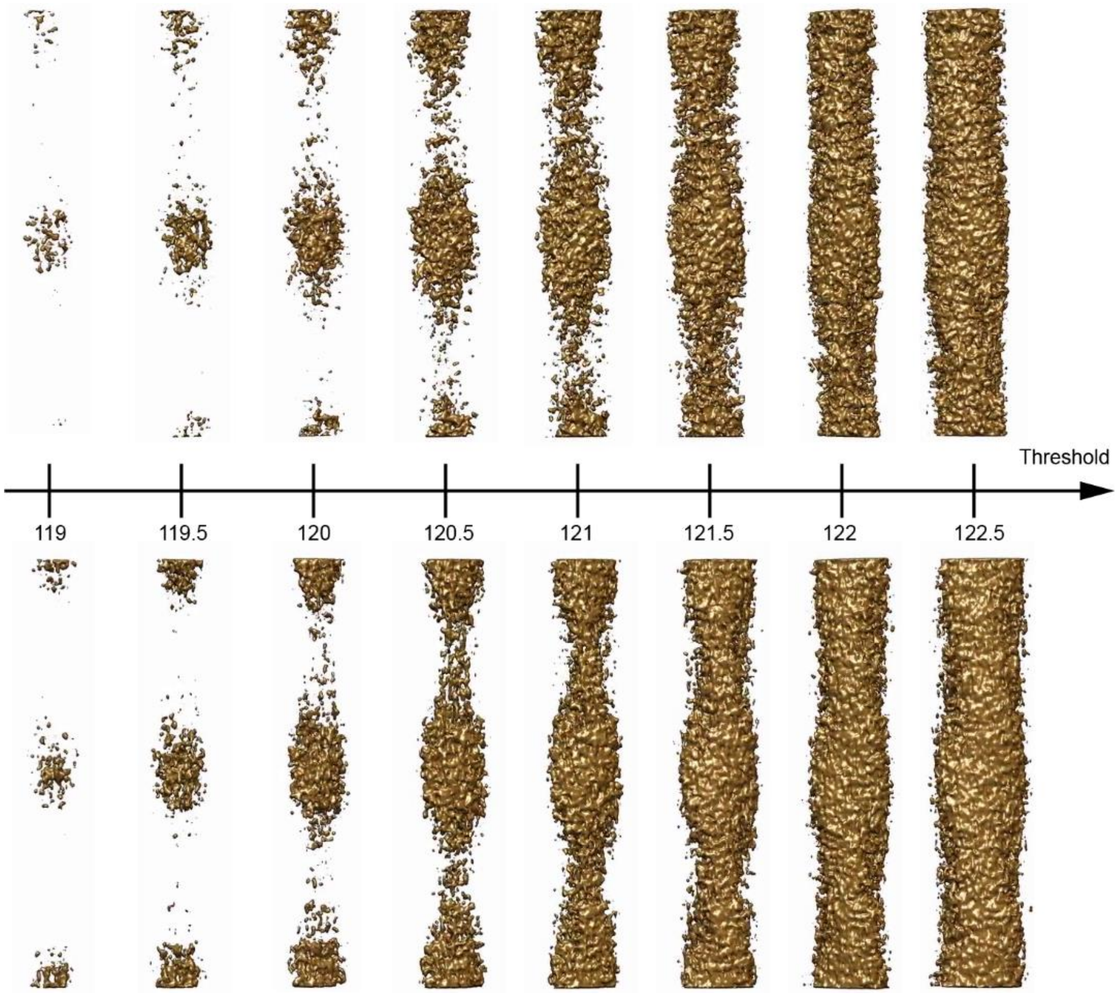

Figure S3. Snapshots of supplementary movie 1. On top, 53.8 nm long subtomograms plotted as 3D volumes without overlap at different thresholds. On bottom, with overlap. Orientation has been chosen to best reflect visualization of wrapping fibers within the CMF.

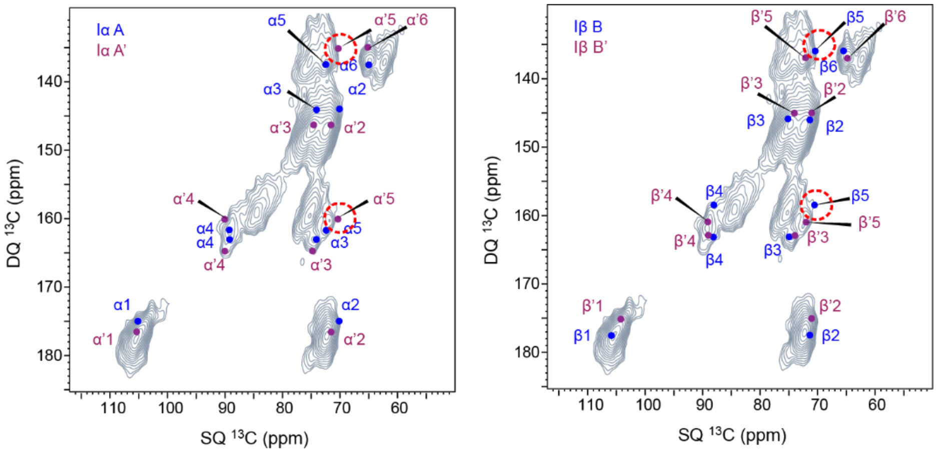

Figure S4. *In-vitro* cellulose microfibril violates the model structure of Iα and Iβ allomorphs. The expected signals are shown as filled dots for the A and A' glucose residues in Iα cellulose and B and B' residues in Iβ cellulose. The expected signals do not follow the spectral pattern. Also, the major violations are highlighted by dash line circles.

Table S1. Parameters used for spectral deconvolution. For i4 signals, both the major and minor components are included. The integral percentages added up to 100.1% due to rounding.

Assignment	Chemical shift (ppm)	Relative Amplitude	Linewidth (ppm)	Integral (%)
i4 (major)	89.2	1.6	2.4	2.4
i4 (minor)	87.6	0.7	1.9	0.8
s^f4	84.7	2.3	2.4	3.6
s^g4	83.3	1.8	2.0	2.3
s4 (minor)	81.5	1.1	2.6	1.9
i3 / s3,5	76.5	3.5	2.5	5.9
i3 / s3,5	74.9	10.1	2.7	18.0
i2,5 / s2	73.5	1.7	2.8	3.2
i2,5 / s2	72.1	17.5	3.5	39.2
i6	64.9	6.1	2.6	10.4
s6	62.4	5.4	3.5	12.4

Table S2. ^{13}C chemical shifts of *in-vitro* CMF. All chemical shifts are on TMS scale. (-): unidentified due to low intensity (e.g., for weak components) or limited resolution (e.g., for some carbon sites with overlapped signals in 1D spectra).

Cellulose form		C1 (ppm)	C2 (ppm)	C3 (ppm)	C4 (ppm)	C5 (ppm)	C6 (ppm)
Chemical shifts from 1D spectrum							
i	major	105.1	72.3	74.6	89.0	72.3	64.8
	minor	-	-	-	87.6 [a]	-	-
s	major	105.1	72.3	74.6	84.5	74.6	62.3
	minor	-	-	-	81.5 [a]	-	-
Chemical shifts from 2D spectra [b]							
i<br>i	major	105.3	72.5	74.8	89.0	72.5	64.7
	minor	-	-	-	87.6	-	-
s	s^f	105.2	72.6	74.6	84.8	74.5	62.3
	s^g	105.2	72.6	74.6	83.2	74.5	62.3

[a] Signals resolved from deconvolution of the 1D spectrum.

[b] Averaged chemical shifts for each carbon sites using the signals identified in refocused INADEQUATE spectrum and off-diagonal peaks assigned in CHHC spectrum (**Tables S3** and **S5**).

Table S3. Observed ^{13}C-^{13}C correlations in refocused INADEQUATE spectrum of unlabeled CMF. DQ chemical shifts are given with a +/- 0.2 ppm peak picking margin.

Spin pair	SQ chemical shifts (ppm)	DQ chemical shift (ppm)
i/s1 – i/s2	105.4 – 72.3	177.5
i/s2 – i/s3	72.3 - 74.7	146.8
i3 – i4	74.6 - 88.9	163.3
i4 – i5	89.2 - 72.7	161.9
i5 – i6	72.4 – 64.8	137.0
s3/5 – s^f4	75.3 - 84.4	159.5
s3/5 – s^g4	73.1 - 83.1	156.3
s5 – s6	75.2 – 62.2	137.2

Table S4. Integration intervals of refocused INADEQUATE spectrum. Uncertainty of the method can be evaluated by considering different ratio calculation methods. In the main text, ratio is calculated from all interior cellulose integrals to surface cellulose integrals.

Site	SQ chemical shift range (ppm)	DQ chemical shift range (ppm)	Absolute integral (/10^4)
i3 in i3 – i4	[75.34 ; 73.33]<br>[76.16 ; 75.35]	[161.76 ; 166.16]<br>[162.79 ; 165.38]	196.2<br>43.4
i4 in i3 – i4	[91.45 ; 88.86]<br>[88.86 ; 86.19]	[163.57 ; 167.19]<br>[163.57 ; 167.45]	124.3<br>50.8
i4 in i4 – i5	[92.58 ; 87.81]<br>[89.99 ; 87.81]<br>[87.81 ; 86.68]	[158.40 ; 163.57]<br>[157.10 ; 158.40]<br>[160.98 ; 163.57]	404.9<br>27.8<br>58.5
i5 in i4 – i5	[72.92 ; 70.90]<br>[72.60 ; 71.22]<br>[73.33 ; 72.92]<br>[72.92 ; 71.71]<br>[71.71 ; 70.66]	[159.17 ; 161.75]<br>[158.39 ; 159.17]<br>[160.47 ; 165.12]<br>[161.75 ; 164.86]<br>[161.75 ; 163.57]	119.3<br>19.0<br>30.4<br>46.5<br>16.6
i5 in i5 – i6	[74.06 ; 71.70]<br>[71.70 ; 68.47]	[128.09 ; 139.75]<br>[130.70 ; 140.27]	890.6<br>377.4
i6 in i5 – i6	[67.26 ; 66.77]<br>[66.77 ; 63.38]<br>[66.77 ; 64.99]<br>[64.99 ; 64.59]<br>[64.59 ; 64.19]<br>[64.19 ; 63.70]	[134.31 ; 138.46]<br>[131.21 ; 137.42]<br>[137.42 ; 140.27]<br>[137.42 ; 139.75]<br>[137.42 ; 139.49]<br>[137.42 ; 138.46]	12.9<br>390.5<br>101.2<br>33.9<br>29.0<br>20.5
s3/5 in s3/5 – s4	[79.23 ; 76.16]<br>[79.07 ; 76.16]<br>[78.42 ; 76.16]<br>[77.94 ; 76.16]<br>[76.16 ; 75.35]<br>[75.35 ; 73.90]<br>[73.90 ; 73.09]<br>[74.37 ; 73.90]<br>[73.09 ; 72.68]<br>[73.09 ; 71.71]<br>[72.68 ; 71.95]	[160.73 ; 164.60]<br>[158.13 ; 160.73]<br>[157.09 ; 158.13]<br>[154.51 ; 157.09]<br>[154.26 ; 162.79]<br>[154.51 ; 161.75]<br>[154.00 ; 160.47]<br>[153.45 ; 154.51]<br>[155.80 ; 158.90]<br>[154.22 ; 155.80]<br>[155.80 ; 158.13]	217.4<br>134.5<br>29.2<br>38.3<br>233.7<br>412.8<br>145.0<br>11.4<br>29.0<br>31.2<br>33.3
s^f4 in s3/5 – s4	[87.81 ; 84.89]<br>[86.68 ; 82.64]	[155.02 ; 160.98]<br>[160.98 ; 163.57]	368.2<br>187.6

	[84.89 ; 84.09]<br>[84.74 ; 84.09]<br>[84.09 ; 83.44]<br>[83.44 ; 82.70]<br>[82.70 ; 80.37]	[154.77 ; 160.98]<br>[152.96 ; 154.77]<br>[157.88 ; 160.98]<br>[157.88 ; 160.98]<br>[157.88 ; 160.98]	184.2<br>14.4<br>89.2<br>75.3<br>107.2
s^g4 in s3/5 – s4	[84.09 ; 80.45]<br>[80.45 ; 79.23]<br>[80.37 ; 79.31]<br>[79.23 ; 78.28]<br>[79.15 ; 78.57]	[152.69 ; 157.88]<br>[153.99 ; 157.35]<br>[157.35 ; 159.45]<br>[154.26 ; 156.58]<br>[156.58 ; 157.88]	394.0<br>62.3<br>26.5<br>24.3<br>6.6
s5 in s5 – i6	[79.21 ; 74.06]	[129.40 ; 140.53]	1083.6
s6 in s5 – i6	[63.38 ; 59.73]<br>[65.40 ; 63.38]<br>[64.99 ; 63.38]<br>[64.59 ; 63.38]<br>[64.19 ; 63.38]<br>[63.70 ; 63.38]	[132.00 ; 142.61]<br>[140.27 ; 148.86]<br>[139.75 ; 140.27]<br>[139.49 ; 140.27]<br>[138.46 ; 139.49]<br>[137.42 ; 139.49]	724.1<br>50.9<br>15.0<br>6.6<br>23.6<br>12.7

Table S5. Observed ^{13}C-^{13}C correlations (off-diagonal) in CHHC spectrum of unlabeled CMF. Denominations are between brackets when uncertain.

Interacting carbon sites	Indirect dimension SQ chemical shifts (ppm)	Direct dimension SQ chemical shifts (ppm)	1-bond (1), multi-bonds (M) and intermolecular (∩) correlations
i6 – s6	63.7	62.5	∩
s6 – i6	62.1	65.3	∩
i6 – i2,5	64.4	72.8	1 (+ M)
s6 – s2	62.4	72.6	M
s6 – s5	62.0	74.2	1
s6 – s3	62.3	75.3	M
i6 – i3	64.1	75.3	M
s6 – s^g4	62.4	83.0	M
i6 – s^g4	65.2	83.7	∩
i6 – i1	64.3	105.9	M
s6 – s1	61.8	105.5	M
i3 – i6	74.8	65.0	M
s3,5 – s6	74.3	62.7	1 (+ M)
s2 – s6	72.9	63.0	M
i2,5 – i6	72.6	65.1	1 (+ M)
i2,5 – i3<br>s2 – s5	72.4	75.0	1 (+ M)<br>M
i3 – i2,5<br>s5 – s2	74.4	72.6	1 (+ M)<br>M
s2 – s^f4	72.5	85.0	M
i2,5 – i4	72.2	88.9	1 (+ M)
s3,5 – s^g4	74.4	83.2	1 + 1
s3,5 – s^f4	74.4	84.8	1 + 1
i2,5 – i1<br>s2 – s1	72.3	105.2	1 (+ M)<br>1
i3 – i1<br>s3,5 – s1	74.4	105.2	M<br>M + M
s^g4 – s2	83.1	72.5	M
s^f4 – s5	84.8	74.6	1
i4 (minor) – i2,3,5	87.6	75.0	1 + 1 (+ M)
i1 – i6	105.4	65.0	M
i1 – i2,5<br>s1 – s2	104.8	73.1	1 (+ M)<br>1
s1 – s3,5<br>i1 – i3	105.3	74.9	M + M<br>M
